# Dynamic optical coherence microscope integrated with cell-cultivation chamber enabled longitudinal and early-stage assessment of tumor spheroid-drug interaction

**DOI:** 10.1038/s41598-026-44296-9

**Published:** 2026-03-19

**Authors:** Ibrahim Abd El-Sadek, Rion Morishita, Yu Guo, Atsuko Furukawa, Pradipta Mukherjee, Shuichi Makita, Masahiro Miura, Satoshi Matsusaka, Yoshiaki Yasuno

**Affiliations:** 1 Computational Optics Group, University of Tsukuba, Tsukuba, Ibaraki 305-8573 Japan; 2 Department of Physics, Faculty of Science, Damietta University, New Damietta City, Damietta, 34517 Egypt; 3 Clinical Research and Regional Innovation, Institute of Medicine, University of Tsukuba, Ibaraki, 305-8575 Japan; 4 Centre for Biomedical Engineering, Indian Institute of Technology Delhi, New Delhi, India

**Keywords:** Biological techniques, Cancer, Medical research, Oncology

## Abstract

We present an integrated system that combines dynamic optical coherence tomography (DOCT) with a small cell-cultivation chamber to enable high-sensitivity and high-temporal-resolution imaging of tumor spheroid’s drug response. Unlike conventional OCT-based volume measurement, which only captures late-stage morphological changes, our system captures early-stage changes in spheroid’s intracellular activity. The compact chamber, positioned beneath the DOCT probe, maintains a cell-culture environment (37 $^\circ$C, 5% CO$_2$) while allowing frequent system access without disturbing the cultivation environment. The proposed system was used to monitor human breast cancer (MCF-7) spheroids response to doxorubicin hydrochloride, tamoxifen citrate, and paclitaxel over 100 hours at 4-hour intervals. While standard OCT-based volume measurement failed to detect early-stage drug concentration impacts, DOCT signals revealed statistically significant differences among the drug concentrations as early as 12 hours. Additionally, high-temporal-resolution imaging at 30-minute intervals over 100 hours revealed rapid and subtle changes in the spheroid morphology and DOCT signals. The results demonstrate that the proposed integrated system of DOCT and cultivation chamber provides a superior, early-readout platform for label-free anti-cancer drug testing compared to traditional structural OCT imaging.

## Introduction

Cancer remains a leading cause of death globally^1,2^. Due to its aggressive nature and resistance to conventional treatments, cancer was responsible for approximately 10 million deaths worldwide in 2020^3^. Tumor spheroids are three-dimensional (3D) clusters of cancer cells that closely mimic the structural and micro-environmental characteristics of *in vivo* tumors^4–6^. Tumor spheroids simulate the 3D architecture of solid tumors and offer significant advantages over animal models, including cost-effectiveness and simplified cultivation protocols with shorter growth cycles^7,8^. Consequently, tumor spheroids are widely utilized in cancer research and for the investigation of anti-cancer drug efficacy^9^.

Tumor spheroids can be evaluated using several conventional techniques, such as histological staining^7,10^, bright-field microscopy^11,12^, and fluorescence microscopy^13,14^. While these methods are widely adopted, most of them are inherently destructive. Fluorescence microscopy requires chemical dyes and histological staining involves tissue sectioning, which precludes their use for continuous longitudinal evaluation. Furthermore, these techniques often suffer from limited penetration depths (typically a few hundred microns) and a lack of robust quantitative capabilities.

In contrast to conventional microscopy, optical coherence tomography (OCT) is a label-free, high-speed imaging modality that provides 3D visualization of biological samples at depths of few millimeters^15^. Due to its non-invasive and volumetric imaging capabilities, OCT can be a powerful tool for the time-course monitoring of tumor spheroids. However, the standard OCT imaging provides only structural information, such as volume measurements, which might not be sufficient for spheroid-drug interaction assessment. It is because volumetric changes are often a “late-stage” indicator of drug-induced cell death. Dynamic OCT (DOCT) has been emerged recently for imaging the intracellular and intratissue activities^16–22^ of various cultured samples, including tumor spheroids^18,20,23,24^ and organoids^25–27^. Compared to the standard OCT, DOCT could be superior for assessing the spheroid-drug interactions. DOCT demonstrated its potential to capture the drug-specific response patterns in human breast cancer (MCF-7) spheroid^23,24^. However, these studies comprised only three treatment time points over 6-days. This low temporal resolution hinders the potential ability of DOCT to capture early-stage intracellular activity changes during spheroid-drug interaction.

To assess both the early (few-hours) and late (few-days) responses of tumor spheroid, longitudinal and frequent tracking of spheroids is required. A few studies have demonstrated longitudinal OCT imaging by placing the OCT probe directly inside an incubator^28,29^. However, the lack of direct access to the OCT system in such configurations makes long-term measurements (spanning several days) inconvenient to perform. Furthermore, any necessary adjustments to the OCT probe within the incubator during the longitudinal measurement may disturb the culture environment and increase the potential for cell contamination.

In this study, we integrated a small cultivation chamber with a DOCT microscope for longitudinal imaging of tumor spheroids. This system allowed for easy and frequent access to the OCT system during long-term (few-days) longitudinal imaging without disturbing the cell-culture environment. Moreover, it enabled spheroid’s tracking over more than four days with an arbitrary temporal-resolution. Longitudinal tracking of MCF-7 spheroids response to doxorubicin hydrochloride (DOX), tamoxifen citrate (TAM), and paclitaxel (PTX) was performed over 100-hours. The results demonstrate that DOCT is superior to standard OCT-based volume measurements for capturing early-stage (as early as 12-hr) drug responses in tumor spheroids.

## Methods

### Integration of DOCT microscope and cell-cultivation chamber

A custom-built swept-source OCT microscope was employed in this study. The system utilized a microelectromechanical system (MEMS)-based sweeping light source (AXP50124-8, Axsun Technologies, Billerica, MA, USA) with a central wavelength of 1310 nm and a scanning speed of 50,000 A-lines/s. The setup incorporated a scan lens (LSM03, Thorlabs Inc., Newton, NJ, USA) with an effective focal length of 36 mm. The axial resolution (in tissue) and lateral resolution were 14 $\upmu$m and 18.1 $\upmu$m, respectively. The optical power incident on the sample was 17 mW. The imaging depth is about 2.9 mm in tissue. It should be noted that, the 1310 nm was chosen for its high penetration depth in turbid medium and it is a well-known light source for the OCT imaging of human skin and anterior segment of the eye. In the field of dermatology OCT systems with wavelength of 1310 nm and power of few to ten few mW are widely used and considered to be safe according to American National Standards Institute (ANSI Z136.1)^30–32^. Longer wavelengths (around 1310 nm) are typically used for anterior segment imaging^33–36^. Since the 1310 nm OCT systems are known to be safe for skin and anterior segment, we believe they are safe for cultured tissue imaging, including spheroid. In the present study, although the spheroids were monitored longitudinally for 100 hours, the OCT beam continuously scans, and hence each position in the sample is exposed for a short time of 362 $\mu$s (i.e. spot size/scanning speed) and revisited after 0.66 s. Thus, we believe that the effective exposure per location is low. Detailed specifications of the OCT device have been reported previously^37^.

**Fig. 1:**
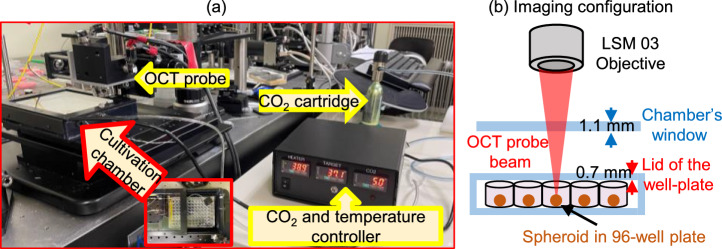
(**a**) Photograph of the integrated DOCT microscope and cell-cultivation chamber. Positioned beneath the OCT probe, the chamber accommodates a 96-well plate containing spheroids, which are supplied with 5 % CO$_2$ from an attached cartridge and maintained at the temperature of 37 $^\circ$C. (**b**) Schematic diagram of the imaging configuration.

Figure 1 shows a photograph of our integrated DOCT microscope. A compact cell-cultivation chamber (SU-141A, BLAST, Japan) with dimensions of 17.5 $\times$ 12.5 $\times$ 3.2 cm$^{3}$ (length $\times$ width $\times$ height) was positioned beneath the OCT probe. The chamber accommodated a 96-well plate containing the spheroids, which was covered with its lid to prevent cell contamination and minimize culture medium evaporation during long-term measurements. This cultivation chamber simulated the environment of a conventional incubator by supplying 5 % CO$_2$ and maintaining a constant temperature of 37 $^\circ$C. In our imaging configuration, the OCT probe beam passes through the 1.1 mm thick glass window of the cultivation chamber and the 0.7 mm thick lid of the well-plate before reaching the spheroid and back-reflecting to the OCT detection system, as schematically illustrated in Fig. 1 (b).

### Dynamic optical coherence tomography (DOCT) imaging

3D DOCT was conducted using our previously established 3D scanning protocol^20^. Briefly, the lateral imaging field of 1 $\times$ 1 mm$^2$ was divided into eight sub-fields, referred to as blocks, each containing 16 B-scan locations. Each block was scanned using a repeated raster scan sequence. The raster scan was repeated 32 times over 6.55 s, capturing 32 frames at each B-scan location with a frame repetition time of 204.8 ms. An OCT volume consisting of 4096 frames captured across 128 B-scan locations was acquired in 52.4 s. The 32-frame sequence at each B-scan location was subsequently analyzed using two DOCT algorithms as follows. The first algorithm employed is the logarithmic intensity variance (LIV). LIV represents the temporal variance of the logarithmic (dB-scaled) OCT intensity time-sequence at each B-scan location^18,20^. LIV quantifies the temporal fluctuations of the backscattered OCT signal that is caused by the alteration of kinematic property of the intracellular scatterers (i.e., intracellular dynamics). When a biological phenomenon, such as hypoxia, induces cell death, it alters the intracellular dynamics, and it is captured by LIV. Our recent investigations demonstrated that LIV is sensitive to the ratio of dynamic scatterers to the total population of dynamic and static scatterers within the coherence volume^38^. Additionally, our recent study demonstrated that the OCT speckle patterns are dramatically altered by random sub-wavelength displacements (such as <100 nm) of the scatterers^39^, and consequently LIV is also sensitive to such tiny motion. The raw LIV volume, consisting of 128 B-scan images, is obtained by applying LIV analysis to the sequential OCT signals captured at each B-scan location. LIV has been described in detail by El-Sadek et al.^18,20^.

The second DOCT algorithm utilized is the late OCT correlation decay speed (OCDS$_l$). To compute the OCDS$_l$, we first computed the auto-correlation of sequentially captured dB-scaled OCT signals at each delay time point. Subsequently, the signal decorrelation curve was obtained as a function of the delay time. The OCDS$_l$ was then calculated as the slope of the auto-correlation decay curve within a specific delay range, such as [204.8, 1228.8 ms]. Recently, we discovered that OCDS$_l$ is sensitive to a specific velocity range of intracellular scatterers^38^. A detailed mathematical description of OCDS$_l$ was provided by El-Sadek et al.^18,20^.

### Image-based quantification

In our previous study^23^, we utilized a semi-automatic segmentation method based on an OCT intensity threshold to segment the spheroid region. However, the bottom surface of the well-plate, which was in contact with the spheroid and exhibited high OCT intensity, was incorrectly segmented as part of the spheroid. This necessitated its manual removal from the final segmentation on a B-scan by B-scan basis. In the present study, we aimed to automate the spheroid segmentation process. Since the well-plate surface is static, it produces a low LIV signal and can be excluded from the final segmentation by incorporating LIV into the segmentation procedure as follows. First, we calculated the product of the OCT intensity and LIV, $\textrm{LIV}(x, y, z) \times \textrm{I}_{dB}(x, y, z)$, where $\textrm{LIV}(x,y,z)$ and $\textrm{I}_{dB}(x,y,z)$ represent the 3D LIV volume and the dB-scaled OCT intensity volume, respectively. (*x*, *y*, *z*) denote the three-dimensional positions within the image.

The obtained image product was subsequently processed using the Eroded Otsu-labeling() function from the py-clesperanto library^40^ (prototype ver. 0.24.1, a GPU-accelerated image processing toolkit for Python). This function performs 3D segmentation and labeling of the image through a sequence of blurring, Otsu-thresholding, and binary erosion. Objects within the resulting eroded image were then identified using connected component labeling. To fill internal voids and eliminate minor artifacts from the initial mask, the resulting mask was processed using the binary_fill_holes() function (Scipy, ver. 1.10.1) followed by the remove_small_objects() function (Scikit-image, ver. 0.21.0). The final 3D segmentation was thus obtained. It is noteworthy that the ability of the proposed method to remove the background region depends on its OCT and LIV levels. In most cases, the segmentation was stable and worked without manual intervention. However, at some late time points, when the culture medium becomes opaque and/or when background noise or floaters with intensity and LIV levels similar to those of the spheroid are present, they may still appear in the final segmentation and must be removed manually. Supplementary Fig. S8 shows representative B-scan images of the OCT intensity, LIV, the $\textrm{LIV}(x, y, z) \times \textrm{I}_{dB}(x, y, z)$ image product, and the final segmentation. The well-plate surface was successfully removed and the segmentation mask included only the spheroid.

The resulting segmentation was subsequently used to calculate the spheroid volume, as well as the mean LIV and mean OCDS$_l$ within the entire spheroid. Additionally, by applying empirically defined cut-offs of 3 dB$^2$ for LIV and 2 $\times 10^{-4}$ ms$^{-1}$ for OCDS$_l$, we determined the tissue volume with LIV values below the threshold (defined as the LIV-based low dynamics volume, or LIV-LDV). The OCDS$_l$-based low dynamics volume (OCDS$_l$-LDV) was calculated using a similar approach. Our previous study^20^ showed that regions with LIV and OCDS$_l$ values below the threshold correlated with propidium iodide (PI) staining of dead cells, whereas regions with higher LIV and OCDS$_l$ values corresponded to viable cells, as confirmed by calcein AM fluorescence imaging. Additionally, our previous study^24^, which used the same spheroid and drug types as in the present work, demonstrated a reasonable pattern correlation between low-dynamics regions and PI-stained areas. Therefore, we believe that LIV-LDV and OCDS$_l$-LDV may reflect the volume of non-viable cells. The computed quantities were then plotted as a function of measurement time for each experimental spheroid condition.

### Volume rendering and time-lapse movies generation

The 3D coordinates of the spheroid center were determined using the aforementioned spheroid segmentation and applied to translate the spheroid to the image center. Subsequently, the shifted OCT, LIV, and OCDS$_l$ volumes were rendered as cut-away views using the Volume Viewer plugin in the Fiji/ImageJ software. These cut-away volume-rendered time-lapse images were then compiled to create movies illustrating the temporal changes in spheroid morphology and DOCT patterns.

### Spheroid preparation and drug treatment protocols

**Fig. 2:**
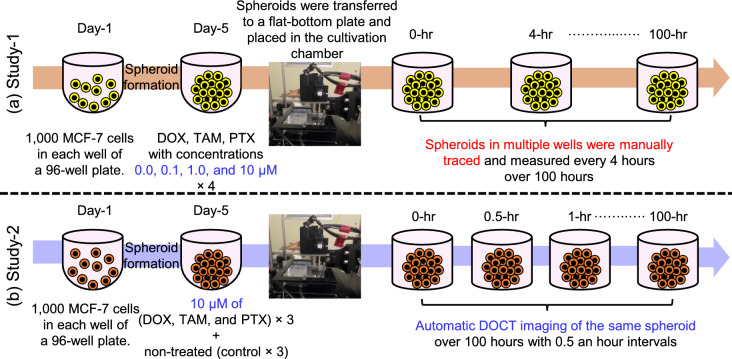
Time chart of MCF-7 spheroid cultivation and imaging. In Study-1, 48 spheroids were treated with DOX, TAM, or PTX on day 5 and imaged in the OCT-integrated chamber over 100 hours at intervals of 4 hours. The first measurement was performed 2 hours after drug administration. In Study-2, 12 spheroids (non-treated, 10 $\upmu$M DOX-treated, 10 $\upmu$M TAM-treated, and 10 $\upmu$M PTX-treated) and three replicates at each case were imaged automatically over 100 hours at intervals of 30 min.

Human breast cancer cells (MCF-7 cell-line) purchased from the Japanese Collection of Research Bioresources (JCRB) cell bank were used in the present study. MCF-7 spheroids were formed by seeding 1000 cells into each well of an ultra-low attachment 96-well plate with a U-shaped bottom and culturing them for five days. The cells were maintained in a cell culture environment under 5% CO$_2$ at 37 $^\circ$C. During the cultivation period, the cells were supplied with a culture medium consisting of a 1:1 mixture of Eagle’s minimal essential medium (EMEM) and F12 (Invitrogen, Waltham, MA, USA) supplemented with 2% B-27 (Invitrogen), 2 ng/mL basic fibroblast growth factor (bFGF; Wako, Osaka, Japan), 2 ng/mL epidermal growth factor (EGF; Sigma-Aldrich, St. Louis, MO, USA), 100 U/mL penicillin G, and 0.1 mg/mL streptomycin sulfate (Wako, Osaka, Japan).

Longitudinal time-lapse imaging of the tumor spheroids comprised two studies, as schematically illustrated in Fig. 2. The first study (Study-1) was conducted to demonstrate comprehensive drug type- and concentration-dependent assessments of spheroid responses using the proposed integrated system. This study involved three drugs, doxorubicin hydrochloride (DOX), tamoxifen citrate (TAM), and paclitaxel (PTX), which were administered at concentrations of 0.1, 1, or 10 $\upmu$M on the fifth day of cultivation. Non-treated spheroids were maintained as a control group. A total of 48 spheroids were included in this study, with four spheroids measured for each treatment condition. Immediately following drug application, the spheroids and 100 $\upmu$L of drug-containing culture medium were transferred to a flat-bottom well plate, placed in the OCT-integrated chamber, and measured longitudinally every 4 hours over a 100-hour period. A flat-bottom well plate was utilized to control the reflection from the well bottom and prevent interference with the OCT imaging. In the present and previous studies^23,24^ we found that the flat-bottom well plate does not significantly induce spheroid movement or drifting, and that the spheroids remain almost stationary during the measurements. The initial measurement was performed 2 hours after drug administration. Due to the manual repositioning of the OCT probe required to measure different spheroids, this study lacked high temporal resolution, where the minimum interval between successive time points was 4 hours.

The second study (Study-2) was performed to demonstrate the high-temporal-resolution imaging capability of the DOCT microscope. In this study, we focused on a single spheroid at a time, which allowed us to fully automate the process and increase the temporal resolution of the time-lapse imaging. This study involved 12 spheroids, specifically non-treated, 10 $\upmu$M DOX-treated, 10 $\upmu$M TAM-treated, and 10 $\upmu$M PTX-treated samples. Three spheroids were imaged under each condition. After drug administration, the spheroid was transferred to a flat-bottom plate, placed in the cultivation chamber, and automatically measured over 100 hours at 30-minute intervals, resulting in 201 DOCT volumes for each sample.

## Results

### Study1: longitudinal imaging and quantification of MCF-7 spheroid response to multiple anti-cancer drugs administered at different concentrations

**Fig. 3:**
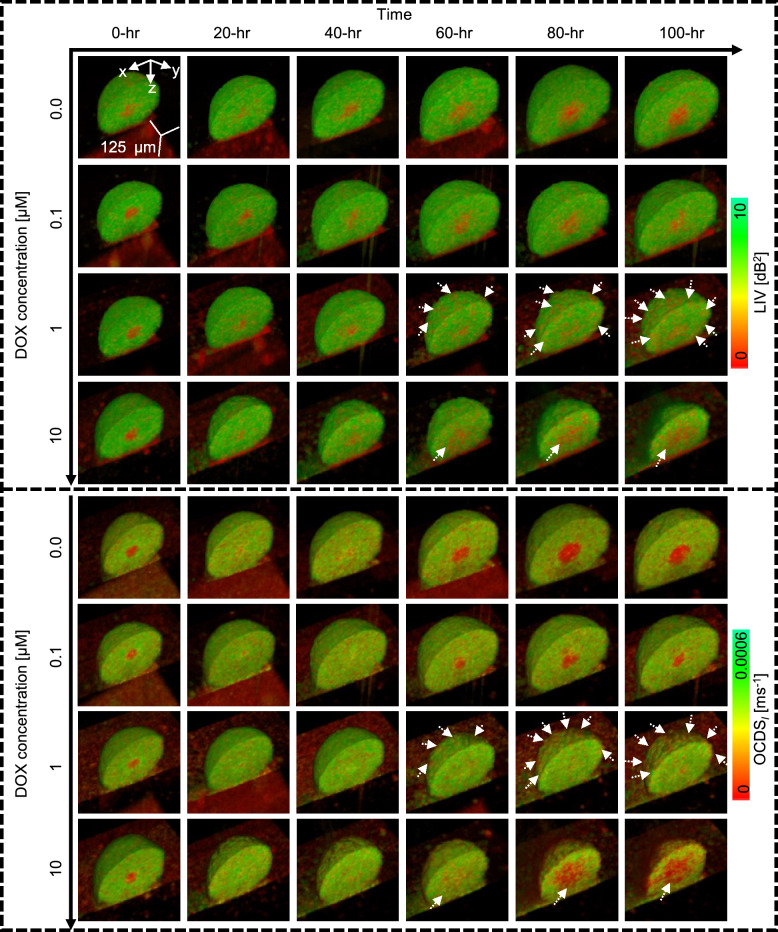
Cut-away volume rendering of LIV and OCDS$_l$ images of non-treated (control) and DOX-treated MCF-7 spheroids. The reduced size of 1 $\upmu$M and 10 $\upmu$M DOX-treated spheroids, along with the low LIV and low OCDS$_l$ signals (red) at the periphery and core (white arrowheads), may reflect the DOX interaction mechanism (see Discussion).

Figure 3 presents representative cut-away volume-rendered LIV and OCDS$_l$ images of non-treated (control) and DOX-treated spheroids at various time points. As demonstrated by the time-course image patterns, increasing the DOX concentration led to a reduction in spheroid size over time. Furthermore, spheroids treated with 1 $\upmu$M DOX exhibited several regions of low LIV and OCDS$_l$ at the periphery [white arrowheads in Fig. 3 (third and seventh rows)], potentially indicating the interaction of DOX with the outer layers of the spheroid. In contrast, at a concentration of 10 $\upmu$M DOX, a region of low DOCT signal was observed at the spheroid center and expanded over time, as indicated by the white arrowheads in [Fig. 3 (fourth and eighth rows)]. These observations may be related to the specific mechanism of DOX interaction (see Discussion).

**Fig. 4:**
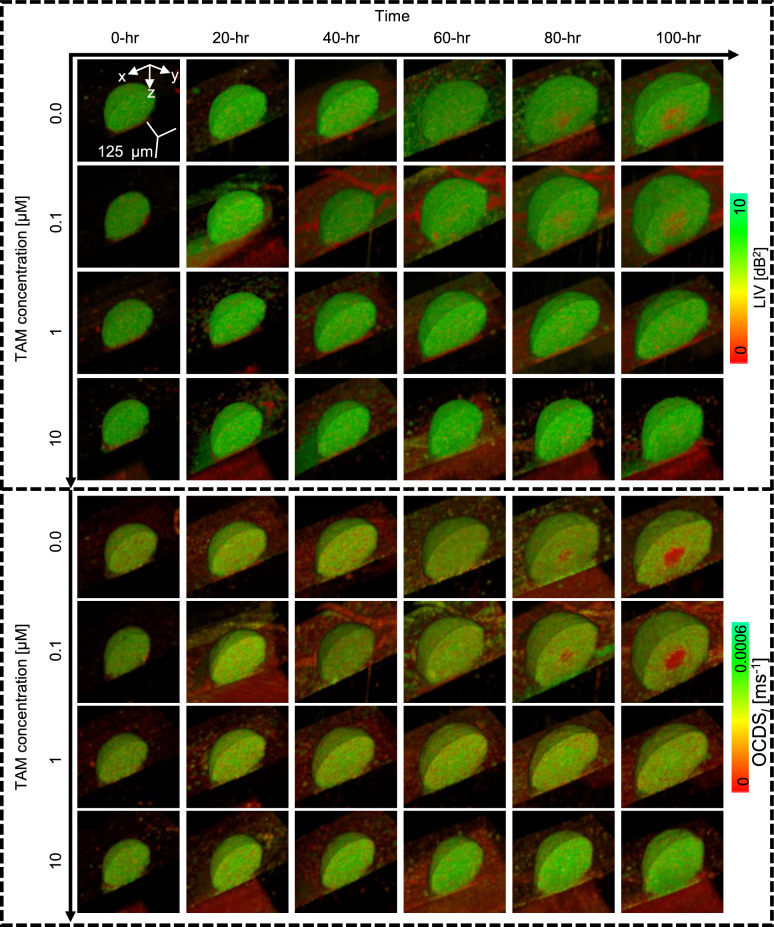
Time-lapse LIV and OCDS$_l$ images of control and TAM-treated spheroids. Although the 1 and 10 $\upmu$M TAM-treated spheroids were smaller than the control, their LIV and OCDS$_l$ appearances showed no significant changes over time, suggesting that TAM did not affect cell viability in these spheroids.

In contrast to the pronounced alterations in DOCT image patterns observed with DOX treatment, TAM-treated spheroids did not exhibit noticeable changes in their LIV or OCDS$_l$ image appearances [Fig. 4]. However, TAM-concentration-dependent changes in the spheroid morphology can be seen. Specifically, higher TAM concentrations lead to smaller spheroid sizes, which may indicate TAM-induced inhibition of cellular proliferation^41,42^, as further elaborated in the Discussion.

**Fig. 5:**
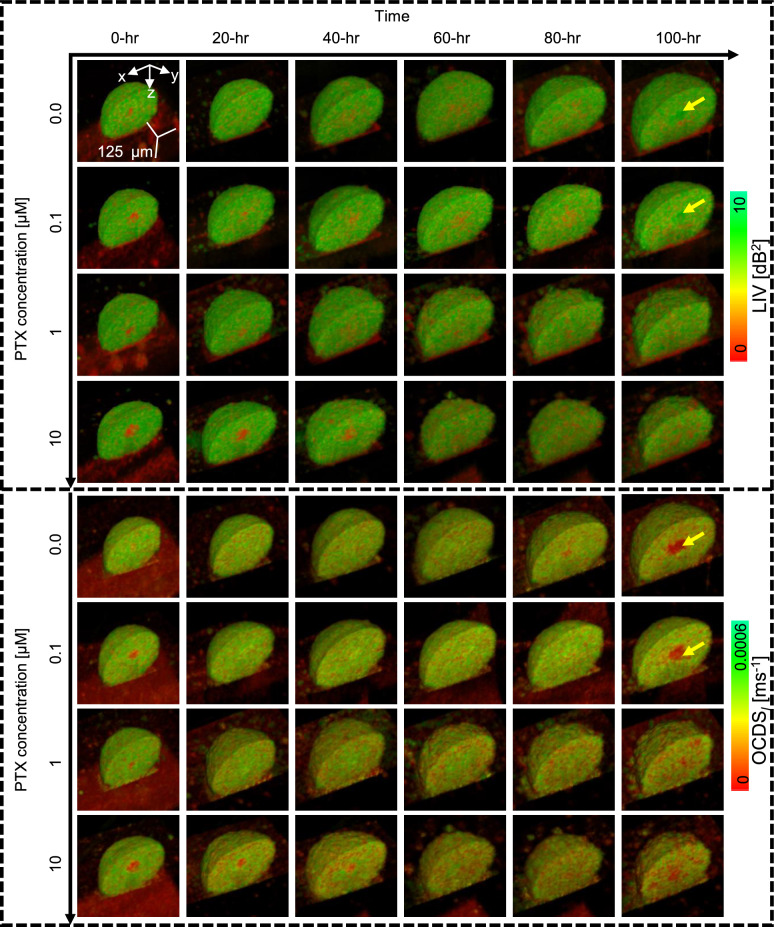
Time-course volume rendering of LIV and OCDS$_l$ of control and PTX-treated spheroids. The increase in the number of low LIV and low OCDS$_l$ spots (red) over time in 1 and 10 $\upmu$M PTX-treated spheroids may be related to the PTX mechanism of interaction (see Discussion).

PTX-treated spheroids [Fig. 5] displayed a mixture of high (green) and low (red) DOCT (LIV and OCDS$_l$) spots, with an increase in the number of low DOCT spots alongside cultivation (treatment) time and PTX concentrations. At later time points, 100-hr, the cores of control and 0.1 $\upmu$M PTX-treated spheroids exhibited higher LIV values compared to their peripheries [yellow arrowheads in Fig. 5 (first and second rows)]. This elevated LIV suggests the presence of a substantial number of moving scatterers at the spheroid center even after 100-hr of incubation. A more comprehensive interpretation of this high LIV is provided in Section 4 of the Supplementary Material.

The full time-lapse movies of the spheroids depicted in Figs. 3, 4, and 5 are available as Supplementary Movies 1–4, 5–8, and 9–12, respectively. Additionally, the *en face* images for these spheroids are summarized in Supplementary Figs. S1-S3. Moreover, additional spheroids measured under each treatment condition for DOX, TAM, and PTX (Supplementary Figs. S5-S7) exhibited similar image patterns to those presented in Figs. 3, 4, and 5.

**Fig. 6:**
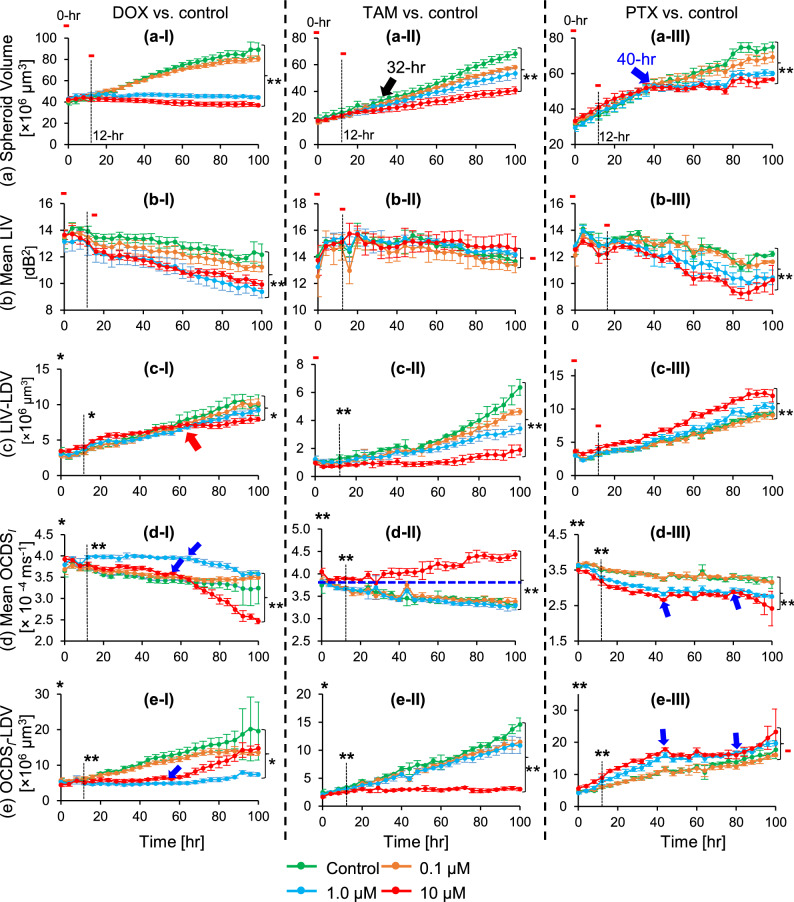
Time-course plots of spheroid volume (**a**), mean LIV (**b**), LIV-LDV (**c**), mean OCDS$_l$ (**d**), and OCDS$_l$-LDV (**e**) of non-treated and drug-treated MCF-7 spheroids. The points and error bars represent the mean and ± SD of 4 samples under each condition, except for the mean and ± SD of the control in the second column plots, which are based on 3 samples. High-temporal-resolution longitudinal imaging using the proposed method enabled identification of the deflection points in the spheroid volume and mean DOCT signals (blue arrows). The marks on the plots indicate the significance of differences in each metric among drug concentrations, as determined by one-way ANOVA at baseline (0 hr), the early time point (12 hr), and the late time point (100 hr). The symbols (-, *, and **) denote $P> 0.05, P <0.05,$ and $P < 0.01$, respectively.

Figure 6 illustrates the time-course alterations in spheroid volume (a), mean LIV (b), LIV-LDV (c), mean OCDS$_l$ (d), and OCDS$_l$-LDV (e) for DOX-, TAM-, and PTX-treated spheroids relative to non-treated controls. The volumetric growth rate of the spheroids [Fig. 6 (a-I)] was significantly suppressed by 1 $\upmu$M and 10 $\upmu$M DOX compared to the control and 0.1 $\upmu$M DOX groups. In contrast, the volumes of TAM- and PTX-treated spheroids [Fig. 6 (a-II) and (a-III)] increased over time across all tested concentrations. However, these spheroids exhibited a concentration-dependent decrease in volume growth rate after 32 and 40 hours of treatment with TAM and PTX, respectively [black and blue arrows in Fig. 6 (a-II) and (a-III)]. Pearson correlation analysis revealed a strong negative and moderate negative correlations between TAM and PTX concentrations and spheroid volume ($\rho = -0.88$ and $-0.657$ for TAM and PTX, respectively), which indicates that higher drug concentrations reduced the spheroid growth rates.

The mean LIV [Fig. 6 (b)] decreased markedly over time following treatment with DOX (b-I) and PTX (b-III); furthermore, the reduction in mean LIV occurred more rapidly at higher concentrations of these drugs. In contrast, no evident concentration- or time-dependent alterations in mean LIV (b-II) can be observed for TAM-treated spheroids.

A clear concentration dependency of LIV-LDV can be observed in TAM-treated spheroids [Fig. 6(c-II)], where higher TAM concentrations resulted in a smaller LIV-LDV. In DOX-treated spheroids (c-I), only after 60-hr [red arrowhead in (c-I)] a DOX-concentration-dependent reduction in LIV-LDV was observed. In contrast, among the PTX-treated spheroids (c-III), only the 10 $\upmu$M concentration displayed an LIV-LDV time-course that differed from the other concentrations.

The means of OCDS$_l$ in control, 0.1 $\upmu$M and 10 $\upmu$M DOX-treated spheroids were similar until 64-hr [Fig.6 (d-I)]. At 10 $\upmu$M DOX, a distinct deflection point in the mean OCDS$_l$ was observed at 56-hr (blue arrowhead). Conversely, the 1 $\upmu$M DOX-treated spheroids exhibited a different mean OCDS$_l$ time-course compared to other DOX concentrations, showing only a slight decrease after 64-hr [blue arrowhead in (d-I)]. In the TAM-treated group [Fig. 6 (d-II)], only the 10 $\upmu$M concentration resulted in a mean OCDS$_l$ time-course distinct from the other TAM concentrations, with a clear separation visible between 10 $\upmu$M and the remaining groups [blue dashed line in (d-II)]. Drug concentration-dependent alterations in mean OCDS$_l$ were observed only in the 1 and 10 $\upmu$M PTX-treated spheroids [Fig. 6 (d-III)]. Specifically, the 10 $\upmu$M PTX treatment resulted in a lower mean OCDS$_l$ than the 1 $\upmu$M treatment at all measured time points. Furthermore, the mean OCDS$_l$ time-course plot for 10 $\upmu$M PTX exhibited two deflection points at 44-hr and 80-hr [blue arrows in (d-III)].

The OCDS$_l$-LDV for spheroids treated with 1 $\upmu$M DOX was markedly smaller than that of the other DOX concentrations [Fig. 6 (e-I)]. Furthermore, a distinct deflection point in the OCDS$_l$-LDV occurred at 56-hr for the 10 $\upmu$M DOX group [blue arrowhead in (e-I)]. In the TAM-treated group (e-II), only the 10 $\upmu$M concentration exhibited an OCDS$_l$-LDV time-course that differed from the other TAM concentrations. In contrast, PTX treatment resulted in a concentration-dependent increase in OCDS$_l$-LDV (e-III). Notably, the 10 $\upmu$M PTX time-course plot featured two specific deflection points at 44-hr and 80-hr [blue arrows in (e-III)].

One-way ANOVA was performed to evaluate differences in spheroid evaluation metrics (spheroid volume, mean DOCT, and LDV) among drug concentrations for each drug type at three time points (0-hr, 12-hr, and 100-hr). Notably, multiple ANOVA tests were performed to evaluate drug-concentration-dependent changes in various metrics, at various treatment time points, and for various drug types. Therefore, these multiple tests were independent, and hence a global correction across all ANOVAs was not applied. For each ANOVA test, when a significant difference was observed, Tukey’s honestly significant difference (HSD) post-hoc test was applied for pairwise comparisons to identify specific concentration pairs at which the spheroid evaluation metrics differed significantly.

At 0-hr, no significant differences among concentrations were observed in spheroid volume, mean LIV, or LIV-LDV for any drug, except for LIV-LDV in the DOX treatment. In this case, one-way ANOVA indicated a significant concentration effect on LIV-LDV values ($P = 0.031$), and Tukey’s HSD post-hoc analysis revealed a significant difference between the control and 0.1 $\upmu$M DOX ($P = 0.047$). In contrast, both the mean OCDS$_l$ and OCDS$_l$-LDV exhibited significant differences among the concentrations of DOX, TAM, and PTX ($P = 0.013, 0.001,$ and 0.003; for mean OCDS$_l$) and ($P = 0.025, 0.034, 0.005$; for OCDS$_l$-LDV). The differences in mean OCDS$_l$ and OCDS$_l$-LDV were significant among control and 10 $\upmu$M DOX and among 0.1 $\upmu$M and 10 $\upmu$M DOX ($P = 0.014, 0.034$ for mean OCDS$_l$ and $P = 0.042, 0.030$ for OCDS$_l$-LDV respectively; Tukey’s HSD test). For TAM, significant difference in mean OCDS$_l$ was found among control and 10 $\upmu$M, and among 1 $\upmu$M and 10 $\upmu$M ($P = 0.009, 0.034$, respectively; Tukey HSD post-hoc). On the other hand, OCDS$_l$-LDV showed significant difference only among control and 10 $\upmu$M ($P = 0.027$). In the case of PTX, significant difference in both the mean OCDS$_l$ and the OCDS$_l$-LDV was found among 0.1 $\upmu$M and 10 $\upmu$M, and among 1 $\upmu$M and 10 $\upmu$M ($P = 0.003, 0.010$ for mean OCDS$_l$ and $P = 0.021, 0.004$ for OCDS$_l$-LDV respectively; Tukey HSD post-hoc). It should be noted that the 0-hr time point was measured 2-hours post drug administration, and hence there might be some drug effect, which was captured be OCDS$_l$ imaging for all the drugs and by LIV-LDV for DOX.

At 12-hr, no significant differences in spheroid volume were observed among concentrations for any drug (DOX: $P = 0.295$; TAM: $P = 0.088$; PTX: $P = 0.230$). In contrast, significant concentration-dependent changes were detected in DOCT-derived metrics, including LIV-LDV for DOX and TAM ($P = 0.026$ and 0.007, respectively), mean OCDS$_l$ for DOX, TAM, and PTX ($P < 0.001$ for all), and OCDS$_l$-LDV for DOX, TAM, and PTX ($P = 0.009, 0.003$, and 0.001, respectively). These findings indicate a higher sensitivity of DOCT for detecting early drug concentration-dependent alterations in spheroids compared with standard OCT-based volume measurements. Tukey’s HSD post-hoc analysis was subsequently performed to identify specific concentration pairs at 12-hr. For DOX-treated spheroids, LIV-LDV differed significantly between 0.1 $\upmu$M and 10 $\upmu$M ($P = 0.030$). Mean OCDS$_l$ showed significant differences between the control and 1 $\upmu$M, between 0.1 $\upmu$M and 1 $\upmu$M, and between 1 $\upmu$M and 10 $\upmu$M ($P< 0.001, P < 0.001$, and $P = 0.005$, respectively), while OCDS$_l$-LDV differed significantly only between 0.1 $\upmu$M and 1 $\upmu$M ($P = 0.011$). For TAM-treated spheroids, LIV-LDV differed significantly only between the control and 10 $\upmu$M ($P = 0.004$). Mean OCDS$_l$ showed significant differences between the control and 10 $\upmu$M TAM, between 0.1 $\upmu$M and 10 $\upmu$M TAM, and between 1 $\upmu$M and 10 $\upmu$M TAM ($P < 0.001$ for all pairs). Additionally, OCDS$_l$-LDV differed significantly between the control and 10 $\upmu$M TAM and between 1 $\upmu$M and 10 $\upmu$M TAM ($P = 0.002$ and 0.026, respectively).

At the late time point (100-hr), both the spheroid volume and DOCT-derived metrics exhibited statistically significant differences among DOX concentrations ($P < 0.001$ for volume, mean LIV, and mean OCDS$_l$, and $P = 0.012$ and 0.010 for LIV-LDV and OCDS$_l$-LDV, respectively). Significant differences in spheroid volume and mean DOCT signals at 100-hr were also observed among TAM concentrations ($P < 0.001$ for volume, mean LIV, mean OCDS$_l$, and OCDS$_l$-LDV) and among PTX concentrations ($P< 0.001, P = 0.005, P < 0.001$, and $P = 0.007$ for volume, mean LIV, LIV-LDV, and mean OCDS$_l$, respectively). Tukey’s HSD post-hoc analysis at 100-hr revealed that spheroid volume differed significantly among all DOX concentration pairs, including between the control and 0.1 $\upmu$M DOX ($P = 0.023$) and between the control and 1 $\upmu$M DOX ($P = 0.049$), with all remaining DOX pairs showing highly significant differences ($P < 0.001$). Significant volume differences were also observed among all TAM concentration pairs ($P < 0.001$), except between 0.1 $\upmu$M and 1 $\upmu$M TAM ($P = 0.121$). For mean LIV, significant differences were detected among several DOX concentration pairs, including the control versus 1 $\upmu$M, control versus 10 $\upmu$M, 0.1 $\upmu$M versus 1 $\upmu$M, and 0.1 $\upmu$M versus 10 $\upmu$M ($P< 0.001, P < 0.001, P = 0.001$, and $P = 0.018$, respectively). In contrast, LIV-LDV differed significantly only between the control and 10 $\upmu$M DOX and between 0.1 $\upmu$M and 10 $\upmu$M DOX ($P = 0.026$ and 0.012, respectively), whereas significant differences were observed among all TAM concentration pairs ($P < 0.001$). Mean OCDS$_l$ showed statistically significant differences for three concentration pairs in both DOX- and TAM-treated spheroids (control vs. 10 $\upmu$M, 0.1 $\upmu$M vs. 10 $\upmu$M, and 1 $\upmu$M vs. 10 $\upmu$M; $P < 0.001$ for all pairs). On the other hand, OCDS$_l$-LDV differed significantly for only one DOX concentration pair (control vs. 1 $\upmu$M DOX; $P = 0.006$), and for five TAM concentration pairs (control vs. 0.1 $\upmu$M, control vs. 1 $\upmu$M, control vs. 10 $\upmu$M, 0.1 $\upmu$M vs. 10 $\upmu$M, and 1 $\upmu$M vs. 10 $\upmu$M; $P = 0.008, 0.002,< 0.001, < 0.001$, and $< 0.001$, respectively).

### Study2: automatic and high-temporal-resolution time-lapse imaging of single spheroids

In Study-1, the temporal resolution was limited to 4-hour intervals, resulting in only 26 time points over the 100-hour imaging period. This low resolution was a consequence of measuring 16 individual spheroids within the 96-well plate at each time point, where the manual targeting required for each well took approximately 15 minutes. To overcome this limitation, we demonstrated high-temporal-resolution imaging (30-minute intervals) by focusing on a single spheroid for each 100-hours session. This 100-hour measurement protocol was repeated twelve times, and a single spheroid was examined in each measurement.

**Fig. 7:**
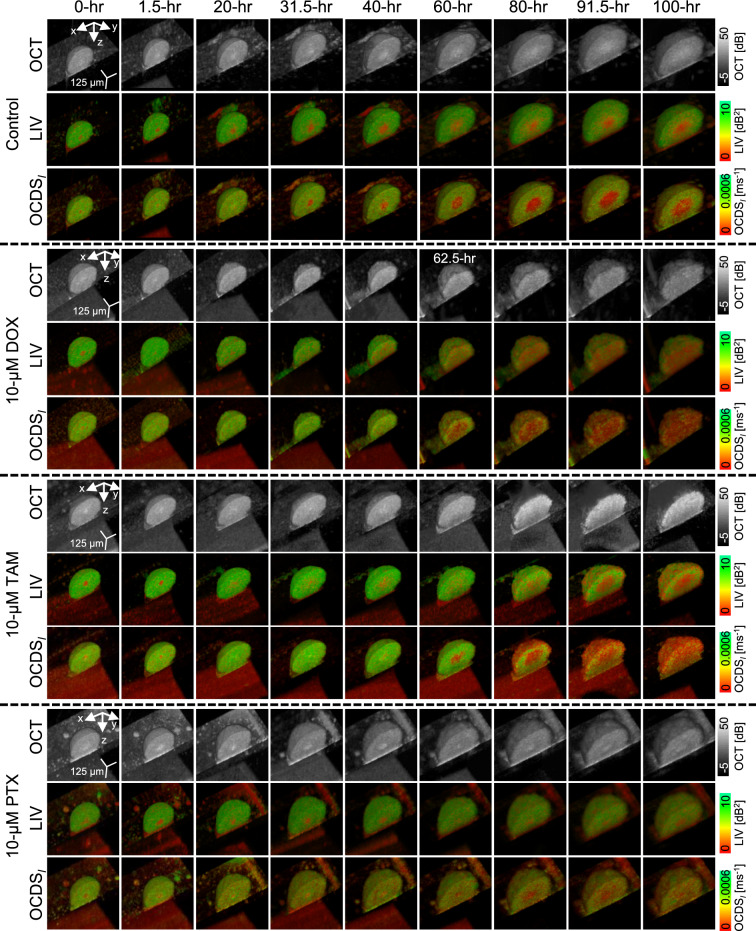
Representative time-course images of non-treated (control), 10 $\upmu$M DOX-treated, 10-$\upmu$M TAM-treated, and 10 $\upmu$M PTX-treated MCF-7 spheroids. The image appearances of control, 10 $\upmu$M DOX-treated, and 10 $\upmu$M PTX-treated spheroids were similar to those in Study-1 [Figs. 3 and 5, respectively]. However, the 10 $\upmu$M TAM-treated spheroid shown here exhibited a marked alterations in the DOCT image appearances, which were not observed at the same TAM concentration in Study-1, as shown in Fig. 4. This difference is discussed in detail in the Discussion.

Figure 7 (first to third rows) presents representative OCT intensity, LIV, and OCDS$_l$ images of a non-treated spheroid, measured automatically over 100 hours at 30-minute intervals. The visual characteristics of these images were consistent with those of the control spheroids observed in Study-1. The complete 100-hour time-lapse movie at 30-minute resolution (Supplementary Movie 13) revealed subtle, fine-scale alterations in the OCT, LIV, and OCDS$_l$ patterns.

The spheroid treated with 10 $\upmu$M DOX [Fig. 7 (fourth to sixth rows)] exhibited swelling over time (refer to Supplementary Movie 14 for the complete 100-hour time-lapse sequence). The low LIV core initially observed at early time points disappeared by 31.5 hr. Notably, the low OCDS$_l$ core vanished even earlier, by 1.5 hr (see Supplementary Movie 14). Subsequently, the low LIV and low OCDS$_l$ (red) at the spheroid center recurred and expanded progressively over time. The high temporal resolution provided by the system developed in this study enabled identifying these specific transition points.

In contrast to the 10 $\upmu$M TAM-treated spheroids presented in Study-1 [Fig. 4], the 10 $\upmu$M TAM-treated spheroid shown in Fig. 7 (seventh to ninth rows) displayed pronounced alterations in its DOCT patterns. Specifically, by 60-hr, several low-LIV spots emerged within the outermost layer of the spheroid. The number of these spots gradually increased over time, eventually forming a distinct low-LIV layer at the spheroid periphery by later time points, such as 100-hr [Fig. 7 (eighth row)]. In addition, a large low-LIV core became visible starting at 85.5 hr (refer to Supplementary Movie 15 for the full time-lapse sequence). Notably, the low-OCDS$_l$ core disappeared at 12-hr but recurred by the 35-hr (as observed in the 40-hr image in Fig. 7 (ninth row) and Movie 15) and expanded progressively thereafter. Furthermore, the spheroid periphery developed several low-OCDS$_l$ spots that increased in number over time, eventually forming a well-defined low-OCDS$_l$ layer. The presence of these low-LIV and low-OCDS$_l$ layers at the periphery may signify the interaction of TAM with the outermost layers of the spheroid. It is important to note that the 10 $\upmu$M TAM-treated spheroids in Study-1 [Fig. 4] did not exhibit such marked alterations in DOCT image appearance compared to the spheroid examined here in Study-2. The potential reasons for this discrepancy are addressed in detail in the Discussion.

The spheroid treated with 10 $\upmu$M PTX [Fig. 7 (tenth to thirteenth rows)] exhibited DOCT patterns similar to those observed for the same PTX concentration in Study-1 [Fig. 5]. Specifically, the low-DOCT core disappeared after 40 hours, after which the spheroid progressively displayed a heterogeneous mixture of high and low LIV and OCDS$_l$ signals. The number of these low-DOCT spots increased over time. The continuous and smooth transition in spheroid morphology and DOCT signals can be observed in the time-lapse movie (Supplementary Movie 16).

The equatorial *en face* images of the control, 10 $\upmu$M DOX-, TAM-, and PTX-treated spheroids depicted in Fig. 7 are summarized in Supplementary Fig. S4.

**Fig. 8:**
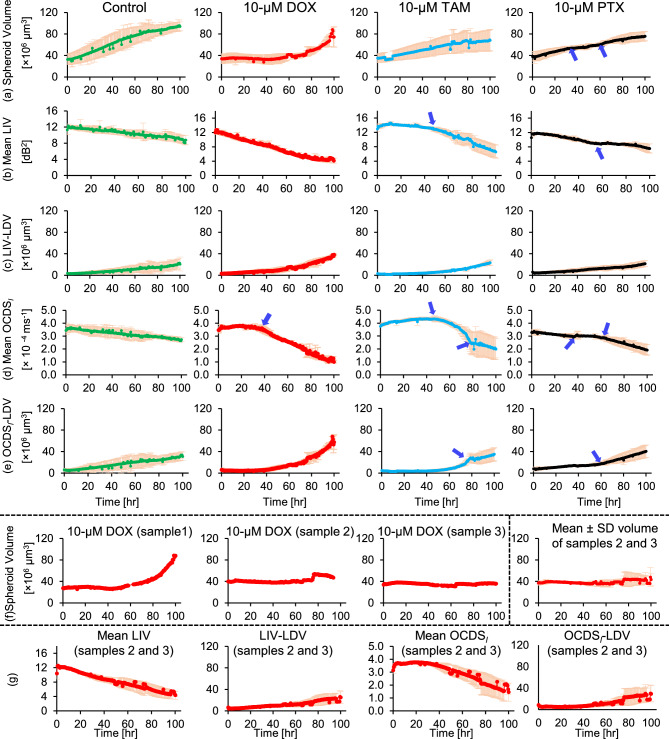
Time-course alterations in spheroid volume (**a**), mean LIV (**b**), LIV-LDV (**c**), mean OCDS$_l$ (**d**), and OCDS$_l$-LDV (**e**) of control, 10 $\upmu$M DOX-, 10 $\upmu$M TAM-, and 10 $\upmu$M PTX-treated spheroids. The dots represent the mean, while error bars represent ± SD among the three measured replicates (n=3). The high temporal resolution (30 min) of the proposed method enabled revealing the subtle and rapid alterations in spheroid evaluation metrics (blue arrows). Although the mean volume of the 3 DOX-treated samples increased at late times, it is mainly because of sample-1 as shown in (**f**). In contrast, the volume growth rate was suppressed in sample-2 and sample-3 (similar to study-1 [Fig. 6 (a-I)]). By excluding sample-1 from the time-course plots, their trends appear comparable to those of the same concentration in study-1 [Fig. 6].

Figure 8 presents the high-temporal-resolution plots for spheroid volume (a), mean LIV (b), LIV-LDV (c), mean OCDS$_l$ (d), and OCDS$_l$-LDV (e) across control, 10 $\upmu$M DOX-, 10 $\upmu$M TAM-, and 10 $\upmu$M PTX-treated groups. Beyond the general trends, which highlight significant differences in volume and mean DOCT signals between control and treated spheroids, the 30-minute temporal resolution revealed rapid alterations in these parameters, as indicated by the blue arrows. As early as 12-hr, mean LIV differed significantly among the control and 10-$\upmu$M TAM-treated spheroids ($P = 0.017$; one-way ANOVA), while significant difference in mean OCDS$_l$ was observed among the control and 10-$\upmu$M PTX-treated spheroids ($P =0.029$; one-way ANOVA). Although the mean volume of the 3 DOX-treated samples increased at late times, it is mainly because sample-1 swelled over time (see individual samples in Fig. 8 (f)). In contrast, in sample-2 and sample-3 the volume growth rate was suppressed (similar to study-1 [Fig. 6 (a-I)]). It should be noted that the replicates in this study (Study-2) were cultivated and measured at different times, because this study tracks a single spheroid in one time-course. Hence, we might have a cultivation environment variability. This may explain the variability of the spheroid volume among samples. The increase in the spheroid volume of DOX-treated spheroid (sample-1) is discussed in the Discussion. Furthermore, by excluding sample 1 from the time-course plots of the 10-$\upmu$M DOX [Fig. 8 (last plot in (f) and the four plots in (g))], they appear comparable to those of the same concentration in study-1 [Fig. 6].

## Discussion

We demonstrate longitudinal DOCT imaging of tumor spheroid drug response by integrating a DOCT microscope with a compact cell-culture chamber. The results revealed that DOCT captures early-stage functional alterations preceding measurable morphological changes. At the baseline (0 hr, measured 2 h post-treatment), spheroid volume did not show significant difference among the drug concentrations, whereas DOCT, specifically OCDS$_l$ and OCDS$_l$-LDV exhibited significant differences among the concentrations for all the drugs [Fig. 6]. This early sensitivity was most pronounced at 12-hr, where spheroid volume remained non-significant across all treatments, while multiple DOCT metrics, including mean OCDS$_l$ and OCDS$_l$-LDV for DOX, TAM, and PTX, and LIV-LDV for DOX and TAM revealed significant concentration-dependent responses [Fig. 6]. These findings highlight the superior ability of DOCT to detect early, dose-dependent drug effects that cannot be recognized by standard OCT-based volume measurements, and indicate that the proposed system might be a powerful tool for early-stage assessment of drug response in tumor spheroids.

In the presented results, we observed distinct dependencies of spheroid morphology and DOCT response patterns on both drug concentration and drug type. These findings could potentially be interpreted by considering the specific mechanisms of the anti-cancer drugs, as detailed below.

DOX is a known inhibitor of topoisomerase II, an enzyme essential for DNA replication. Consequently, its primary action is the suppression of cellular proliferation^43–45^. Furthermore, DOX-induced inhibition of topoisomerase II leads to double-stranded DNA breaks, which ultimately trigger apoptosis^43–45^. We hypothesize that the marked suppression of spheroid growth observed at concentrations of 1 $\upmu$M and 10 $\upmu$M DOX [Fig. 6 (a-I)] is likely attributable to the inhibition of cell proliferation. Meanwhile, the observed reductions in mean LIV [Fig. 6 (b-I)] and mean OCDS$_l$ [Fig. 6 (d-I)] in DOX-treated spheroids suggests a possible correlation between DOCT signal reduction and the DOX-induced apoptotic cell death. Further validation of the low DOCT signals observed in DOX-treated spheroids using apoptosis-specific markers such as Annexin V is planned for future studies.

TAM is known to suppress the proliferation of MCF-7 cells by inducing cell cycle arrest^46,47^. Specifically, TAM arrests cells in the G1 phase, a stage dedicated to RNA and protein synthesis in preparation for division, thereby preventing the transition into the S phase (the DNA replication phase). By inhibiting this progression, TAM effectively slows overall cellular growth. We hypothesize that the observed reduction in the growth rate of TAM-treated spheroids relative to the control [Fig. 6 (a-II)] is possibly associated with this cell cycle inhibition. Interestingly, however, the mean LIV and mean OCDS$_l$ across the four measured spheroids [Fig. 6 (b-II) and (d-II)] showed no concentration-dependent changes. We suspect that under the conditions of this specific experiment, TAM did not alter the spheroid’s intracellular dynamics detectable by our DOCT. While the exact reason for this relative ineffectiveness remains an open question, the result was consistently observed across all the replicates in this study.

PTX is known to stabilize microtubule (MT) dynamics and prevent their depolymerization^48,49^. Consequently, PTX treatment leads to cell-cycle and mitotic arrest. Furthermore, because microtubules serve as the essential platform for intracellular transport, the stabilization of MTs by PTX may halt these transport processes. The observed reduction in the growth rate of PTX-treated spheroids after 40-hr [Fig. 6 (a-III)] may relate to the inhibition of mitosis. In contrast, the reduction in mean LIV and mean OCDS$_l$ [Fig. 6 (b-III and d-III)] may relate to the impairment of intracellular transport through the MTs. Moreover, the increased number of low DOCT spots [Fig. 5] over time suggests a spatial variation in cell viability, potentially indicating localized regions of cell death within the spheroid. Our previous studies^23,24^ showed that low DOCT regions correlated with dead (PI-stained) cells observed in fluorescence images of PTX-treated spheroids.

By comparing the results of Study-1 and Study-2; several consistencies as well as some inconsistencies were identified as discussed in detail in the following four paragraphs.

The DOCT image appearances of the non-treated, 10-$\upmu$M DOX-treated, and 10-$\upmu$M PTX-treated spheroids were consistent across both studies, suggesting good reproducibility of the DOCT imaging of the spheroid response to these drugs. Additionally, the DOCT metrics, particularly the mean OCDS$_l$, were able to detect early differences (at 12-hr) between the non-treated and PTX-treated spheroids in both studies, which may indicate the ability of DOCT to identify early changes during spheroid-drug interaction. Although the response patterns of the 10-$\upmu$M TAM-treated spheroids differed between the two studies, the DOCT metrics (mean OCDS$_l$ in Study-1 and mean LIV in Study-2) showed significant differences between the non-treated and TAM-treated spheroids at the early time point (12-hr) in both studies.

In Study-1, the growth of 10 $\upmu$M DOX-treated spheroids was markedly suppressed over time [Fig. 6(a-I)], likely due to the inhibition of cellular proliferation as previously discussed. In contrast, one measured sample of 10 $\upmu$M DOX treated spheroids in Study-2 [sample-1 in Fig. 8 (f)] showed a marked volume increase, even though it was under the same condition as in Study-1 and other samples measured with the similar protocol [samples 2 and 3 in Fig. 8 (f)]. It is well-known that DOX uptake^50^ and release^51^ within spheroids are temperature-dependent. The small cultivation chamber used in this study has a less stable temperature and CO$_2$ than standard (large) cultivation chambers. Temperature and CO$_2$ records over 24 hours showed that the temperature sometimes drops to 35.2 $^\circ$C and fluctuates around, and some other times the temperature remains stable at about37 $^\circ$C. On the other hand, the CO$_2$ was fluctuating between 4.5 to 6 % (see Section 5 in the Supplementary Material). Since the measured samples in Study-2 were seeded and measured at different times, not at the same time as in Study-1, we speculate that slight temperature and CO$_2$ instability in our cultivation chamber may promote different interactions between the spheroids and DOX, resulting in the above difference. However, a direct relationship between slight variation in the chamber’s temperature and the variability of spheroid responses to drugs cannot be conclusively established. Furthermore, spheroid growth is inherently sensitive to mechanical^52–54^, biochemical^55^, and environmental factors^56,57^. Such instability may further influence how a spheroid responds to DOX. One of the mechanisms of DOX is the generation of free radicals that damage cell membranes^44^. We suspect that this mechanism may have influenced DOX-treated spheroid (sample-1) of Study-2 [Fig. 8 (f)]. Specifically, it is possible that DOX-generated free radicals led to membrane rupture and a subsequent degradation of intercellular adhesion. This loss of structural integrity may have led to the observed swelling and volume increase.

Spheroids treated with 10 $\upmu$M TAM in Study-1 maintained consistently high LIV and OCDS$_l$ signals throughout the 100-hour period [Fig. 4], suggesting that the drug did not affect the viability of those particular MCF-7 spheroids. In contrast, under similar treatment concentration, the spheroid in Study-2 developed a distinct peripheral layer characterized by low LIV and low OCDS$_l$ signals [Fig. 7 (eighth and ninth rows)]. While the specific impact of temperature on TAM uptake remains under-research, we speculate that minor temperature fluctuations within the cultivation chamber and the possible instability of spheroid growth might have facilitated increased drug uptake or altered its interaction mechanism with the cells. Given that several studies have demonstrated TAM’s ability to induce apoptosis in MCF-7 cells^47,58–60^, the low-LIV and low-OCDS$_l$ signals observed at the periphery in Study-2 are hypothesized to reflect TAM-induced apoptosis. This interpretation, however, remains speculative based solely on DOCT data; future investigations employing apoptosis-specific markers, such as Annexin V, would be instrumental in validating this localized drug effect.

In addition to the possible temperature fluctuations of the cultivation chamber, it should be noted that during Study-1, the OCT probe was manually moved to scan sample by sample at each time-point, while in Study-2 the OCT beam was stationary on the same well over 100-hours. Hence, the OCT-light-induced heat effect is investigated and it was found to be negligible and no heat accumulation over time. The detailed analysis can be found at Section 6 in Supplementary Material.

In the present study, we used a 1310 nm OCT device with scanning speed of 50,000 Alines/s. This wavelength lies in the near-infrared region, where photochemical and phototoxic effects are significantly lower than those at visible wavelengths^61,62^. Each position in the sample is exposed for a short period of 362 $\mu$s. Moreover, previous studies reported that OCT imaging does not induce significant changes in DNA integrity, motility, or cellular functions^63^, and is suitable for sensitive biological applications such as embryo imaging^64,65^ and developmental biology studies^66^ due to its low phototoxicity. Consistent with these findings, the control spheroids in our study showed no morphological damage or abnormal changes in typical DOCT appearance over time, indicating negligible cumulative photobiological effects under the present conditions.

Our previous DOCT-based investigations of tumor spheroid drug responses were limited to a pseudo-longitudinal study (PLS) design. In practice, a PLS is cross-sectional; different spheroids are measured at each specific time point and subsequently discarded. This is because the spheroids must be removed from their cultivation environment, stained with fluorescent dyes for validation, and then transported to the OCT laboratory for OCT imaging, and hence they are no longer suitable for subsequent measurements^24^. In contrast, the high-temporal-resolution longitudinal DOCT imaging system proposed here offers three distinct advantages. First, because measurements occur during cultivation, samples are never removed from their controlled environment, eliminating disturbances caused by sample transfer. Second, by tracking the same individual spheroid throughout the experiment, the study is less susceptible to inter-sample biological variations. Third, this approach provides a high density of time points, facilitating a fine-scale time-lapse analysis. A comparison between our previously demonstrated PLS^24^ and the proposed longitudinal imaging of 10 $\upmu$M DOX-treated spheroids is provided in Section 7 of the Supplementary Material. It highlights the advantages of the proposed method over the PLS.

## Conclusion

We demonstrated longitudinal and high-temporal resolution DOCT imaging of tumor spheroid drug response during cultivation over more than four days. As early as 12 hours, DOCT signal revealed statistically significant differences among the drug concentrations, compared to the conventional OCT-based volume measurement. In conclusion, the proposed longitudinal DOCT platform might serve as a powerful imaging modality for the early-stage assessment of tumor spheroid-drug interactions and drug development.

## Supplementary Information

Supplementary Information 1.

Supplementary Information 2.

Supplementary Information 3.

Supplementary Information 4.

Supplementary Information 5.

Supplementary Information 6.

Supplementary Information 7.

Supplementary Information 8.

Supplementary Information 9.

Supplementary Information 10.

Supplementary Information 11.

Supplementary Information 12.

Supplementary Information 13.

Supplementary Information 14.

Supplementary Information 15.

Supplementary Information 16.

Supplementary Information 17.

## Data Availability

Correspondence and requests for materials should be addressed to Yasuno.
