# Supplementary material for: Dynamic optical coherence microscope integrated with cell-cultivation chamber enabled longitudinal and early-stage assessment of tumor spheroid-drug interaction
Source: Sci Rep. 2026 Mar 19;16:14254. doi: 10.1038/s41598-026-44296-9 (PMC13139614; doi:10.1038/s41598-026-44296-9)
Supplement: Supplementary file 17 — Supplementary Information 17. [file 41598_2026_44296_MOESM17_ESM.pdf]

Supplementary material

Dynamic optical coherence microscope integrated with cell-cultivation chamber enabled longitudinal and early-stage assessment of tumor spheroid-drug interaction

Ibrahim Abd El-Sadek[1,2], Rion Morishita[1], Yu Guo[1], Atsuko Furukawa[3], Masahiro Miura[1], Shuichi Makita[1], Pradipta Mukherjee[1,4], Satoshi Matsusaka[3], and Yoshiaki Yasuno[1]

[1]*Computational Optics Group, University of Tsukuba, Tsukuba, Ibaraki 305-8573, Japan*

[2]*Department of Physics, Faculty of Science, Damietta University, New Damietta City, 34517, Damietta, Egypt*

[3]*Clinical Research and Regional Innovation, Institute of Medicine, University of Tsukuba, Ibaraki 305-8575, Japan*

[4]*Centre for Biomedical Engineering, Indian Institute of Technology Delhi, New Delhi, India*

This supplementary material contains four figures (Figs. S1–S4) comprising *en face* cross-sectional images of the spheroids presented in the main manuscript. Additionally, Figs. S5–S7 show extra spheroids at each concentration of DOX, TAM, and PTX measured alongside those shown in Figs. 3–5 of the main manuscript. These additional spheroids exhibit similar image appearances to those presented in the main manuscript.

In addition, some insight about elevated LIV at the spheroid core in some spheroid cases, temporal fluctuations in the temperature and CO_2 levels supplied by our small cultivation chamber, OCT-light-induced heat analysis, and comparison of longitudinal and pseudo-longitudinal imaging were discussed in Sections 3-7, respectively.

1. *En face* cross-sections of the spheroids presented in the main manuscript

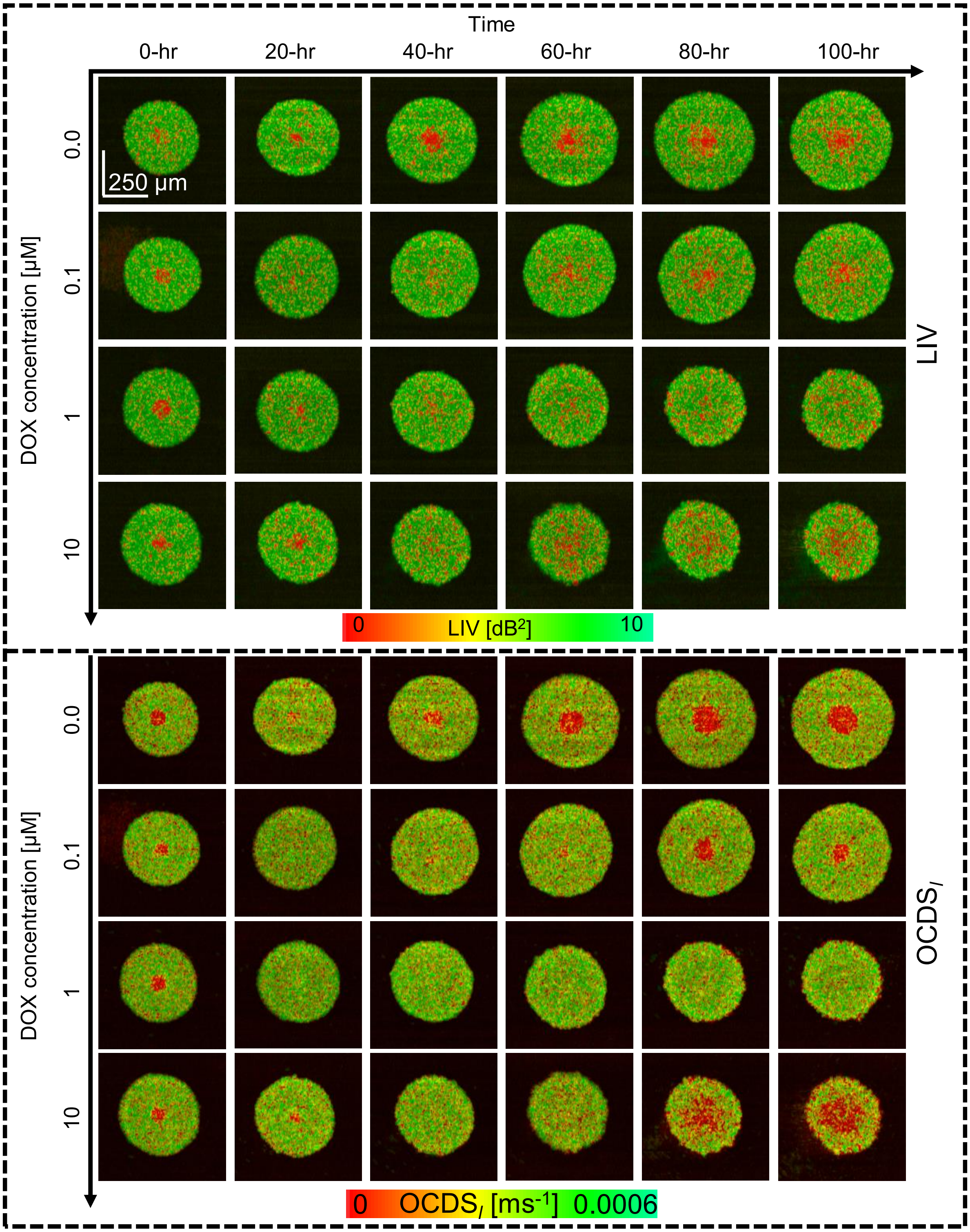

Figure S1: En face cross-sectional LIV and OCDS$_l$ images of control and DOX-treated MCF-7 spheroids. The images were extracted from the spheroid volumes presented in Fig.3 of the main manuscript.

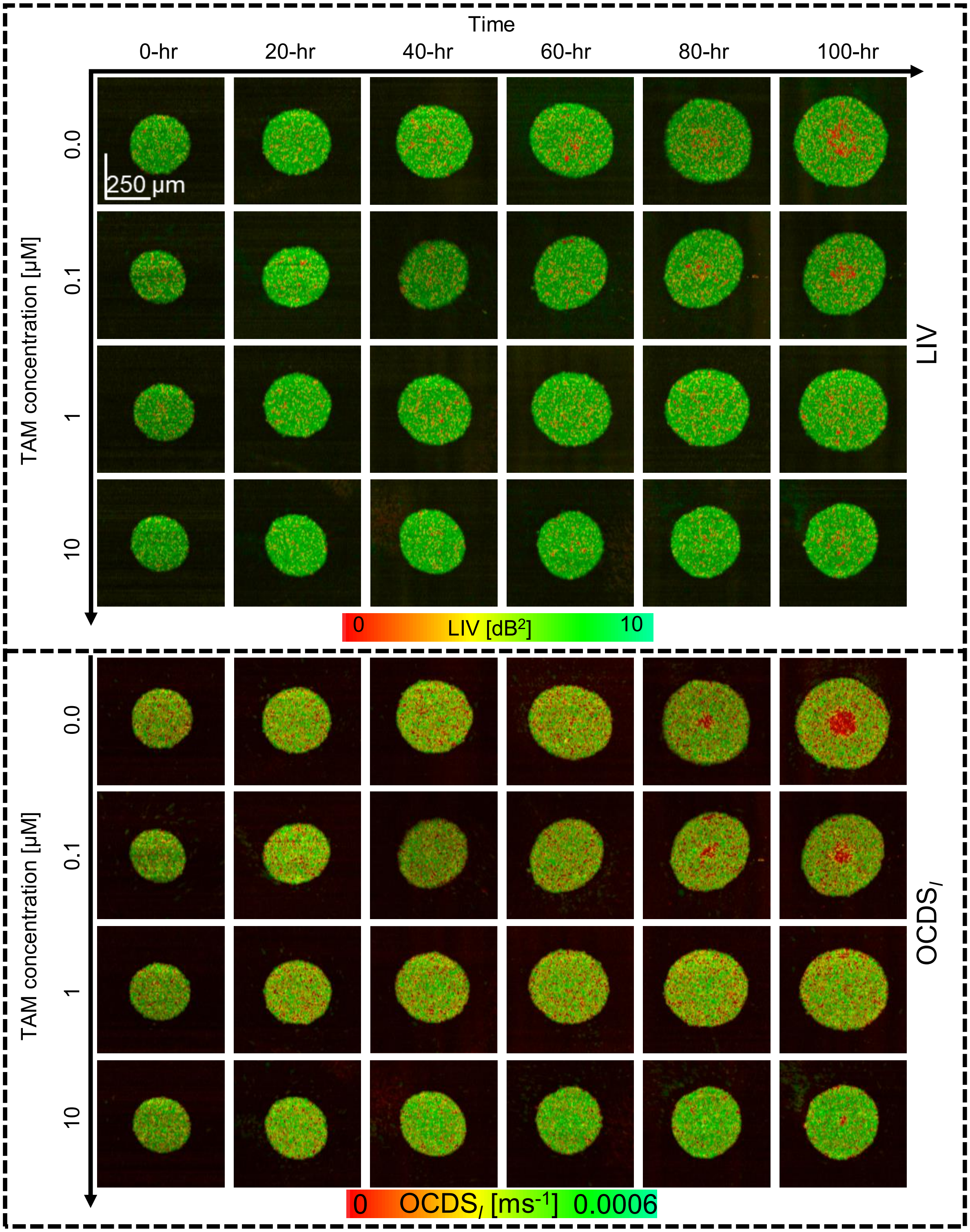

Figure S2: En face cross-sectional LIV and OCDS$_l$ images of control and TAM-treated spheroids presented in Fig.4 of the manuscript.

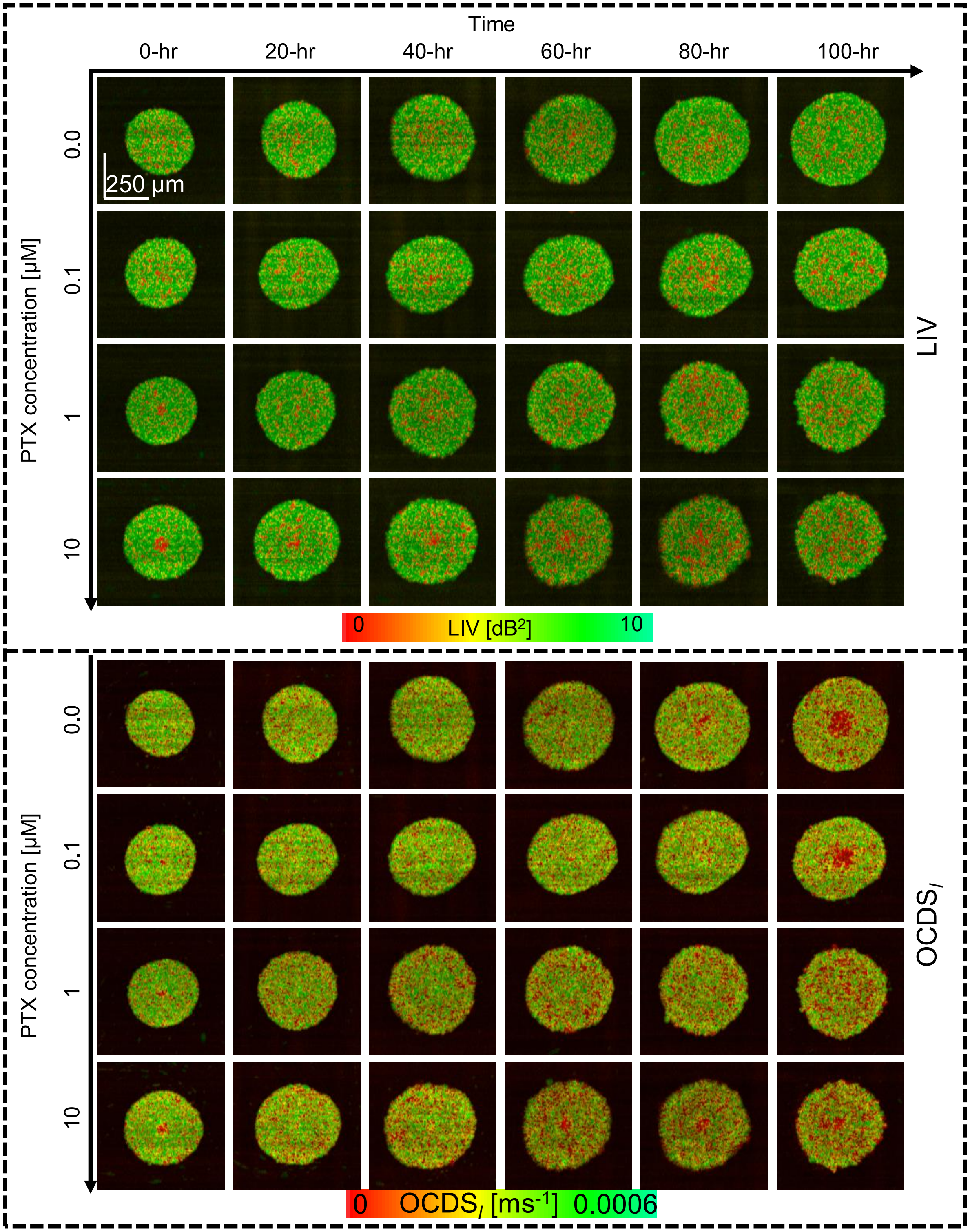

Figure S3: En face cross-sectional LIV and OCDS$_l$ images of control and PTX-treated spheroids presented in Fig.5 of the manuscript.

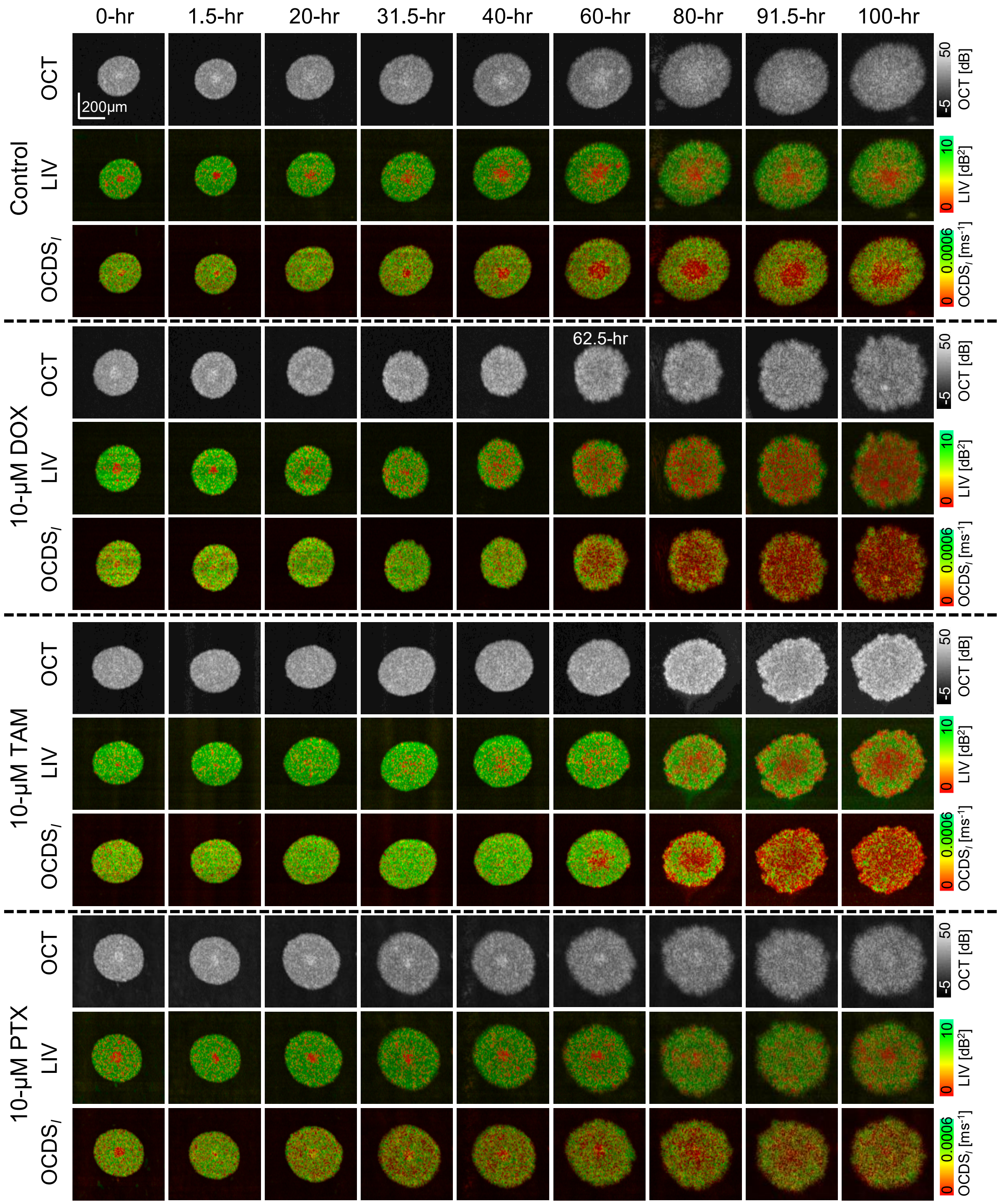

Figure S4: Equatorial *en face* OCT, LIV and OCDS$_l$ images of control, and 10 μM DOX-, TAM-, and PTX-treated spheroids presented in Fig. 7 of the main manuscript.

2. Additional spheroid cases measured along with those presented in Study-1 of the manuscript

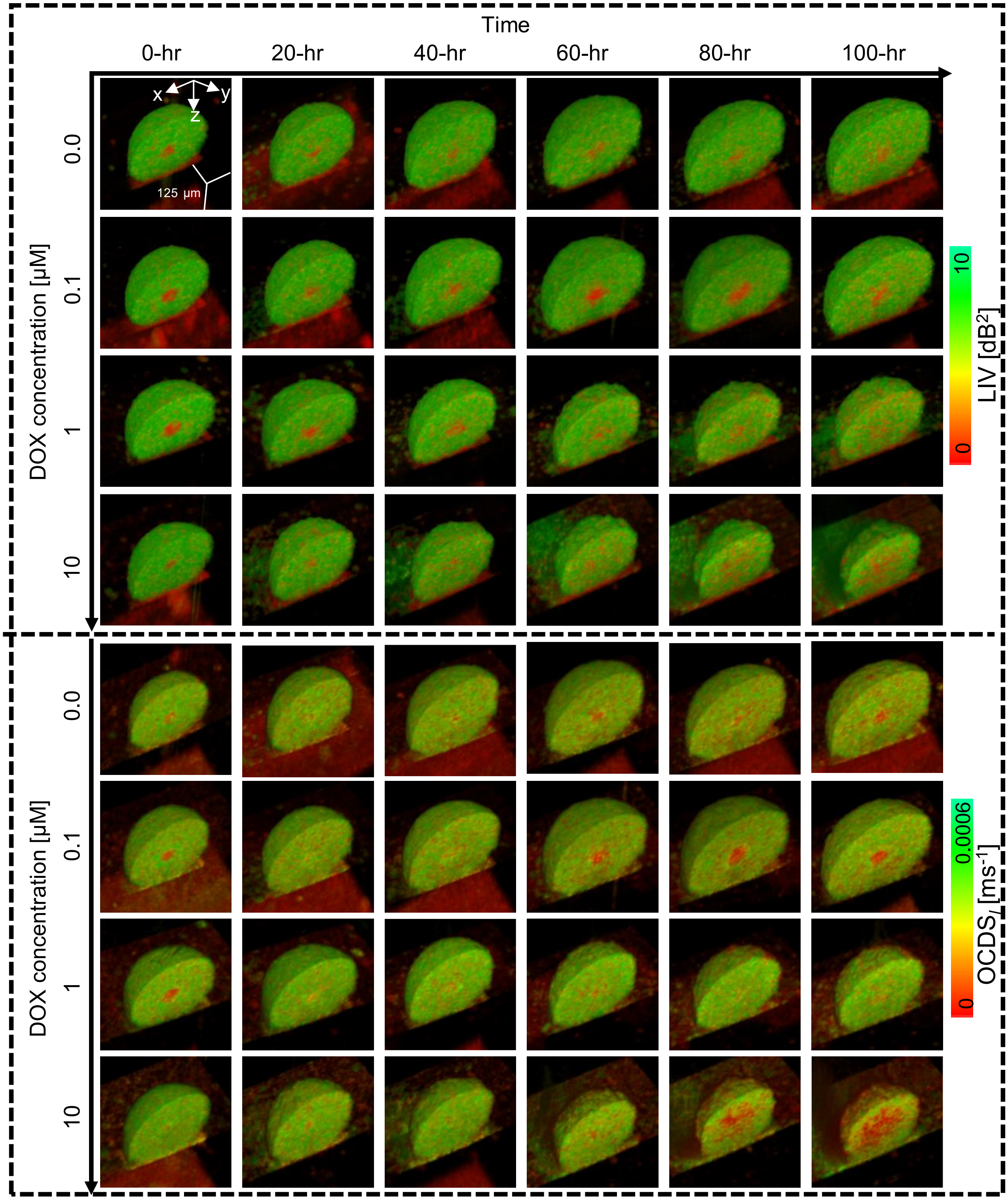

Figure S5: Additional spheroid measured at each concentration of DOX. The spheroids exhibit similar image appearances to those presented in Fig. 3 of the main manuscript.

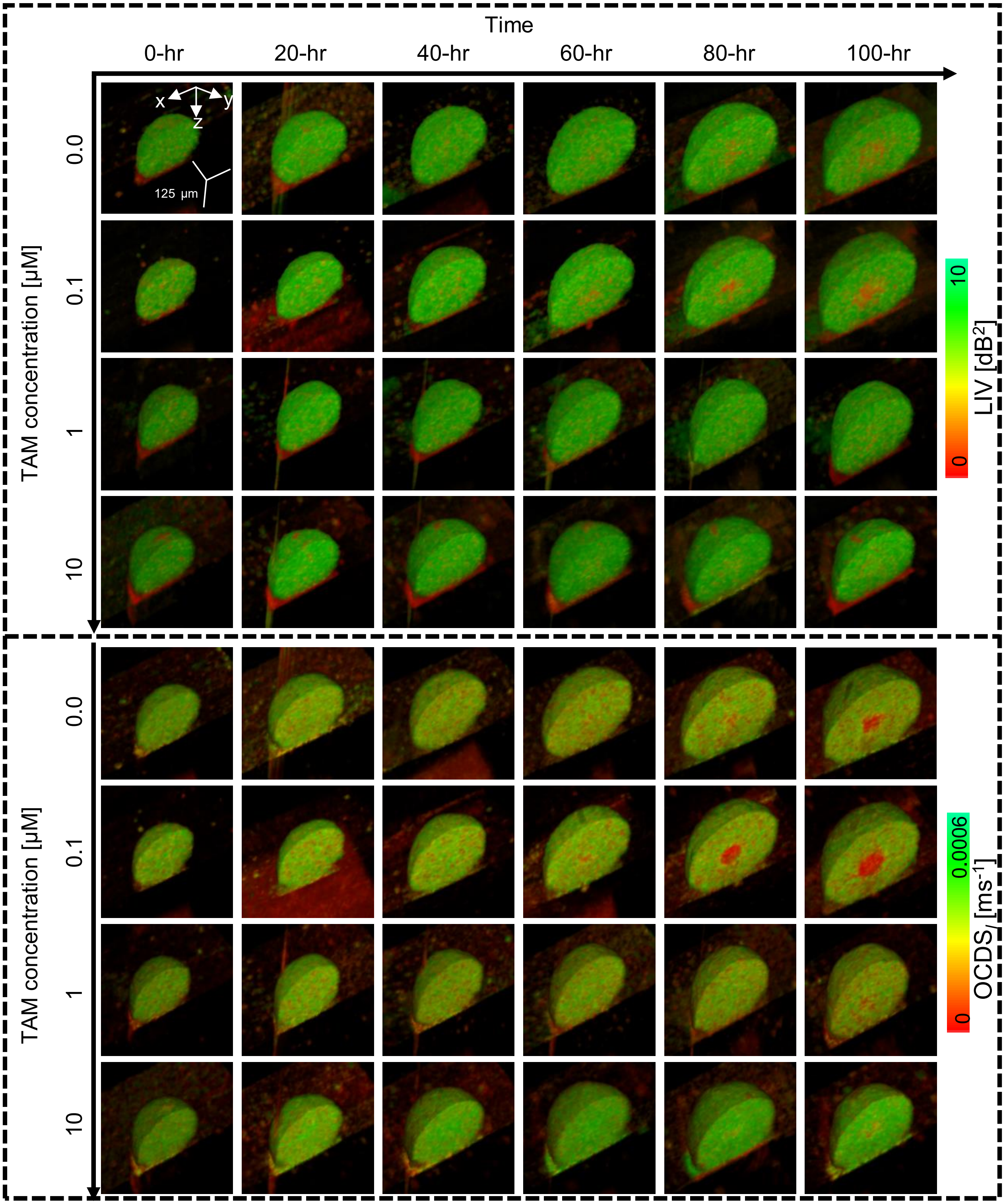

Figure S6: Extra spheroid measured at each concentration of TAM. The spheroids exhibit similar image appearances to those presented in Fig. 4 of the manuscript.

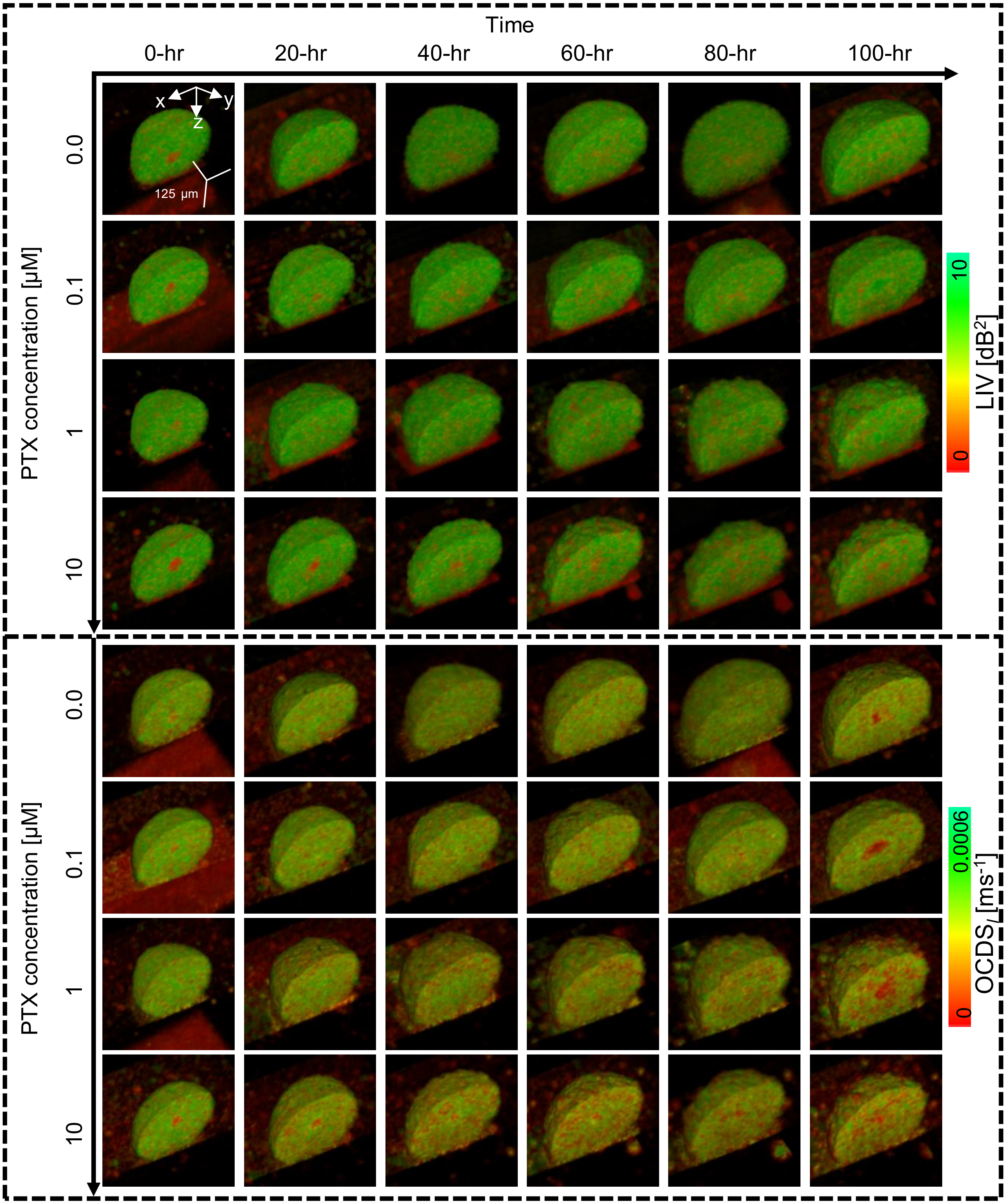

Figure S7: Extra spheroid measured at each concentration of PTX. The spheroids exhibit similar image appearances to those presented in Fig. 5 of the manuscript.

3. Spheroid segmentation

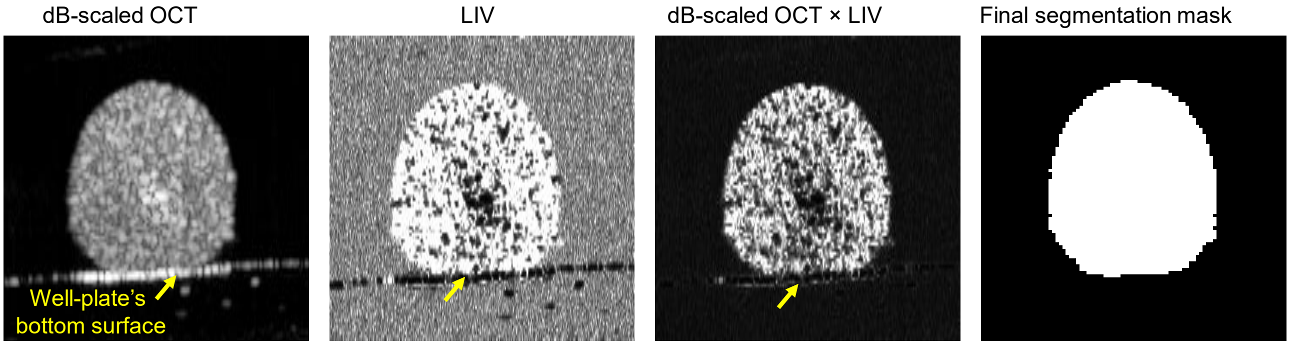

Figure S8: Representative B-scan image of the spheroid's OCT intensity including the well-plate's bottom surface beneath the spheroid, LIV, LIV and intensity image product, and final segmentation. The well plate surface was successfully removed and only the spheroid was well segmented.

4. Insight into elevated LIV at the core after 100 hours incubation via the new DOCT algorithm

Recently, we developed two novel DOCT algorithms, namely authentic LIV (aLIV) and swiftness, which reflect the occupancy of moving intracellular scatterers over all scatterers and their motion speed, respectively[1]. Figure S8 shows the aLIV and swiftness computed from the same raw data of control and 0.1 \uM PTX-treated spheroids at 100-hr presented in Fig. 6 (first and second rows) in the main manuscript. In both cases, the spheroid core exhibited both high aLIV and high swiftness (green). These results may indicate that the spheroid core has a large number of dynamic scatterers (high aLIV) that are moving fast (high swiftness).

The spheroid core is well-known to undergo necrosis owing to nutrient and oxygen deprivation[2,3]. During necrosis, the cell membrane forms blebs and eventually loses its integrity, resulting in leakage of cellular components into the extracellular space [4,5]. We hypothesize that during this process, cellular components leaked into the surrounding environment move randomly, and their motion contributes to the high aLIV and high swiftness observed in the spheroid core.

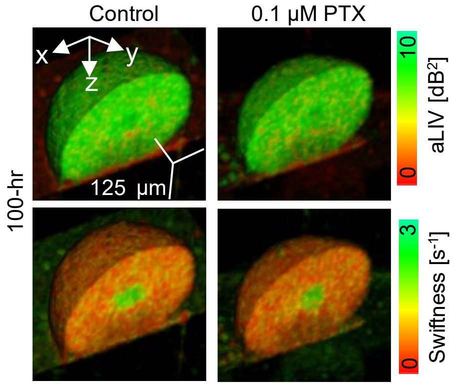

Figure S9: aLIV and swiftness volume rendered images generated from the same raw data of control and 0.1 μM PTX-treated spheroids at 100-hr presented in Fig.6 in the manuscript. The high aLIV at the spheroid center suggests the presence of a large number of dynamic scatterers that are moving rapidly (high swiftness).

5. Temporal fluctuations in the temperature and CO_2 levels supplied by our small cultivation chamber

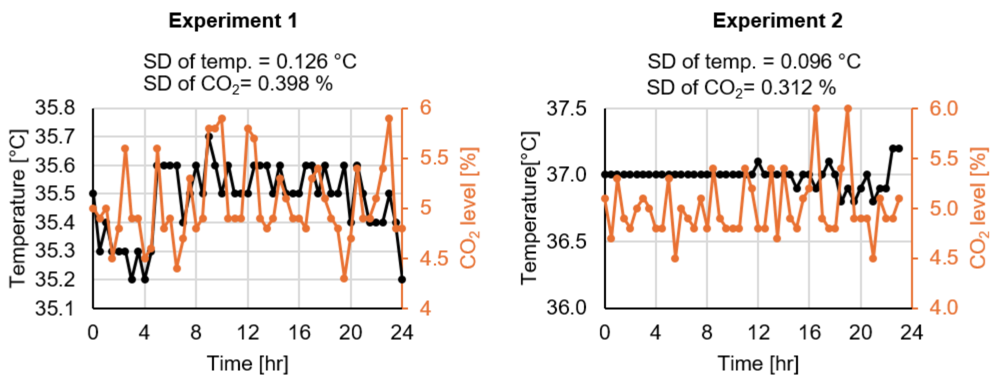

Figure S10: Temporal fluctuations in temperature and CO_2 levels provided by the small cultivation chamber. It should be noted that this temperature measure is not a direct measure of the spheroid temperature, but it is the measure of the top surface of the well-plate, where the thermocouple is attached.

6. OCT-light-induced heat analysis

By considering the system and measurement parameters (wavelength 1310 nm, incident power 17 mW, and scanning area 1 × 1 mm²), and assuming water as the primary constituent of the culture medium (absorption coefficient $\mu \approx 50\ \mathrm{m^{-1}}$ at 1310 nm), the steady-state temperature rise (ΔT) induced by OCT illumination can be estimated. The absorbed optical power in the medium was calculated using Beer–Lambert's law:

$$P_{abs} = P_{inc}(1 - e^{-\mu z}), \tag{1}$$

where z is the optical path length through the liquid. For a liquid volume of 100 µL, the effective depth was approximated as 2 mm, yielding Pabs1.61 mW. Assuming steady-state heat conduction in water and approximating the scanned region as a localized heat source with an effective radius $r = \sqrt{(A/\pi)}$ mm (corresponding to the 1 × 1 mm² scan area), the temperature rise can be estimated using the steady-state solution for a localized heat source in a homogeneous medium given by Baffou and Quidant [6] as:

$$\Delta T = \frac{P_{abs}}{4\pi k r}, \tag{2}$$

where k is the thermal conductivity of water (k= 0.6 W/m.K). Substituting these values yields of ΔT of 0.38 °C.

Because the spheroid was fully immersed in the culture medium and imaged over time scales much longer than the thermal diffusion time (on the order of seconds), the spheroid temperature is expected to closely follow the surrounding medium temperature. Therefore, the estimated temperature rise of ~ 0.38 °C represents a conservative upper bound for the temperature increase experienced by the spheroid. Moreover, since the OCT beam scans only a 1 × 1 mm² region within a well plate containing a substantially larger liquid volume, the surrounding medium acts as an effective heat sink, further mitigating local temperature accumulation. Consequently, the actual temperature rise at the spheroid is expected to be lower than this estimate. Nevertheless, eliminating any potential laser-induced thermal effects by implementing a beam shutter during the 30-minute interval between successive measurement time points may be considered in future system designs.

7. Comparison between our previous pseudo-longitudinal study and the proposed longitudinal study

Figure S11 shows a comparison of the time course of the proposed high-temporal-resolution longitudinal imaging (red) and the previous PLS [7] (blue) of 10 μM DOX-treated spheroids. As is evident in the OCDS$_l$ images [Fig. S11(a)], the pseudo time course in the PLS comprises only three time points. This is because at each measurement time point, different samples need to be cultivated and measured, and increasing the number of time points requires more and more samples. In contrast, using the proposed longitudinal imaging, we longitudinally measured the same spheroid at 201 time points over 100 h. The figure shows representative data at 9 time points, and the full cut-away volume rendered time-lapse movie is provided as Supplementary Movie 15.

The comparison of the spheroid volume plots [Fig. S11 (b)] revealed a significant departure between the two studies. In the PLS, as the spheroids measured at different treatment times were not the same individual spheroid, the consistency of the measured spheroid volume among the time points might be disturbed. Because the proposed method traced the same sample over days, it was free from such inconsistencies. Similarly, the mean LIV of the PLS did not show a clear tendency [Fig. S11 (c)], and it was higher than that of the proposed longitudinal study at 72-hr. This high mean LIV can be attributed to inter-sample variation. In addition, it might be related to an artificially high LIV signal caused by dissociated cells during sample transfer. In contrast, the proposed method is free from such disturbances, and it clearly revealed a reduction in the mean LIV related to DOX-induced apoptosis [8,9].

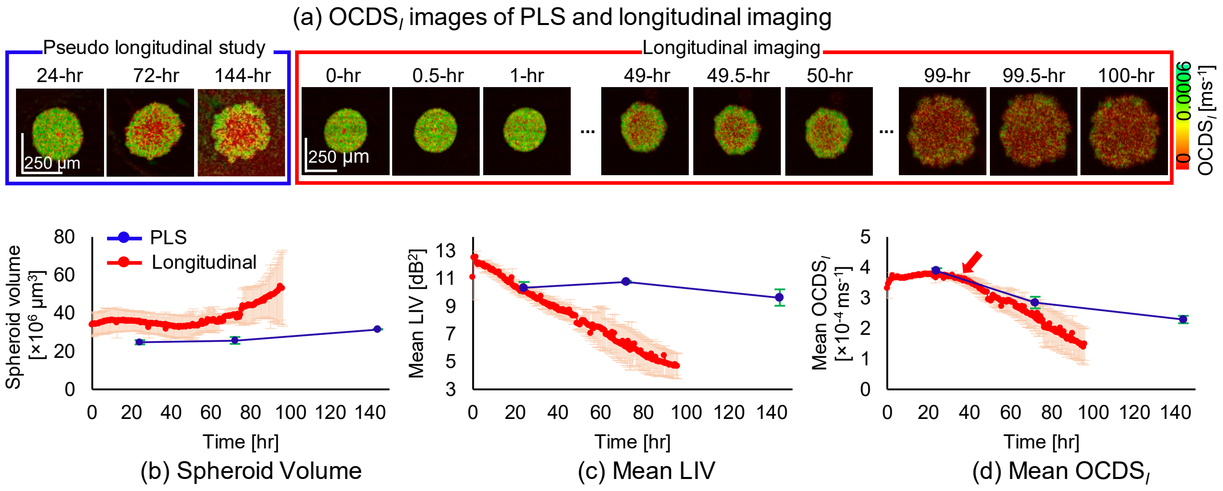

Figure S11: Comparison of OCDS$_l$ images (a), spheroid volume (b), mean LIV (c), and mean OCDS$_l$ (d) of 10 μM DOX-treated spheroids between the previous pseudo-longitudinal study (PLS) and proposed high-temporal-resolution longitudinal study. The plots of the longitudinal study (red dots and lines) are reprinted from Fig. 8 in the main manuscript, while those of the PLS are reprinted from Ref. [7].

References

1. R. Morishita, P. Mukherjee, I. A. El-Sadek, T. Seesan, T. Mori, A. Furukawa, S. Fukuda, D. Lukmanto, S. Matsusaka, S. Makita, and Y. Yasuno, "Dynamic optical coherence tomography algorithm for label-free assessment of swiftness and occupancy of intratissue moving scatterers," Biomed. Opt. Express, BOE **17**(1), 322–345 (2026).
2. E. C. Costa, A. F. Moreira, D. de Melo-Diogo, V. M. Gaspar, M. P. Carvalho, and I. J. Correia, "3D tumor spheroids: an overview on the tools and techniques used for their analysis," Biotechnology Advances **34**(8), 1427–1441 (2016).

3. R. Mukomoto, Y. Nashimoto, T. Terai, T. Imaizumi, K. Hiramoto, K. Ino, R. Yokokawa, T. Miura, and H. Shiku, "Oxygen consumption rate of tumour spheroids during necrotic-like core formation," Analyst **145**(19), 6342–6348 (2020).
4. G. Majno and I. Joris, "Apoptosis, oncosis, and necrosis. An overview of cell death.," Am J Pathol **146**(1), 3–15 (1995).
5. S. Y. a Proskuryakov, A. G. Konoplyannikov, and V. L. Gabai, "Necrosis: a specific form of programmed cell death?," Experimental Cell Research **283**(1), 1–16 (2003).
6. G. Baffou and R. Quidant, "Thermo-plasmonics: using metallic nanostructures as nano-sources of heat," Laser & Photonics Reviews **7**(2), 171–187 (2013).
7. I. Abd El-Sadek, R. Morishita, T. Mori, S. Makita, P. Mukherjee, S. Matsusaka, and Y. Yasuno, "Label-free visualization and quantification of the drug-type-dependent response of tumor spheroids by dynamic optical coherence tomography," Sci Rep **14**(1), 3366 (2024).
8. N. Pilco-Ferreto and G. M. Calaf, "Influence of doxorubicin on apoptosis and oxidative stress in breast cancer cell lines," Int J Oncol **49**(2), 753–762 (2016).
9. S. Pengnam, S. Plianwong, P. Patrojanasophon, W. Radchatawedchakoon, B. Yingyongnarongkul, P. Opanasopit, and P. Charoensuksai, "Synergistic Effect of Doxorubicin and siRNA-Mediated Silencing of Mcl-1 Using Cationic Niosomes against 3D MCF-7 Spheroids," Pharmaceutics **13**(4), 550 (2021).
